# New Insights into Autoinducer-2 Signaling as a Virulence Regulator in a Mouse Model of Pneumonic Plague

**DOI:** 10.1128/mSphere.00342-16

**Published:** 2016-12-14

**Authors:** Eric C. Fitts, Jourdan A. Andersson, Michelle L. Kirtley, Jian Sha, Tatiana E. Erova, Sadhana Chauhan, Vladimir L. Motin, Ashok K. Chopra

**Affiliations:** a Department of Microbiology and Immunology, University of Texas Medical Branch at Galveston, Galveston, Texas, USA; b Department of Pathology, University of Texas Medical Branch at Galveston, Galveston, Texas, USA; Swiss Federal Institute of Technology Lausanne

**Keywords:** Yersinia pestis, quorum sensing, autoinducer-2, virulence, pneumonic plague, animal models, transcriptomics

## Abstract

The *Enterobacteriaceae* family members, including the infamous *Yersinia pestis*, the causative agent of plague, have a highly conserved interbacterial signaling system that is mediated by the autoinducer-2 (AI-2) quorum-sensing molecule. The AI-2 system is implicated in regulating various bacterial virulence genes in diverse environmental niches. Deletion of the gene encoding the synthetic enzyme for the AI-2 substrate, *luxS*, leads to either no significant change or, paradoxically, an increase in *in vivo* bacterial virulence. We showed that deletion of the *rbsA* and *lsrA* genes, components of ABC transport systems that interact with AI-2, synergistically disrupted AI-2 signaling patterns and resulted in a more-than-50-fold decrease in *Y. pestis* strain CO92 virulence in a stringent pneumonic plague mouse model. Deletion of *luxS* or *lsrK* (encoding AI-2 kinase) from the Δ*rbsA* Δ*lsrA* background strain or complementation of the Δ*rbsA* Δ*lsrA* mutant with the corresponding gene(s) reverted the virulence phenotype to that of the wild-type *Y. pestis* CO92. Furthermore, the administration of synthetic AI-2 in mice infected with the Δ*rbsA* Δ*lsrA* Δ*luxS* mutant strain attenuated this triple mutant to a virulence phenotype similar to that of the Δ*rbsA* Δ*lsrA* strain in a pneumonic plague model. Conversely, the administration of AI-2 to mice infected with the Δ*rbsA* Δ*lsrA* Δ*luxS* Δ*lsrK* mutant did not rescue animals from lethality, indicating the importance of the AI-2–LsrK axis in regulating bacterial virulence. By performing high-throughput RNA sequencing, the potential role of some AI-2-signaling-regulated genes that modulated bacterial virulence was determined. We anticipate that the characterization of AI-2 signaling in *Y. pestis* will lead to reexamination of AI-2 systems in other pathogens and that AI-2 signaling may represent a broad-spectrum therapeutic target to combat antibiotic-resistant bacteria, which represent a global crisis of the 21st century.

**IMPORTANCE** *Yersinia pestis* is the bacterial agent that causes the highly fatal disease plague. The organism represents a significant concern because of its potential use as a bioterror agent, beyond the several thousand naturally occurring human infection cases occurring globally each year. While there has been development of effective antibiotics, the narrow therapeutic window and challenges posed by the existence of antibiotic-resistant strains represent serious concerns. We sought to identify novel virulence factors that could potentially be incorporated into an attenuated vaccine platform or be targeted by novel therapeutics. We show here that a highly conserved quorum-sensing system, autoinducer-2, significantly affected the virulence of *Y. pestis* in a mouse model of pneumonic plague. We also identified steps in autoinducer-2 signaling which had confounded previous studies and demonstrated the potential for intervention in the virulence mechanism(s) of autoinducer-2. Our findings may have an impact on bacterial pathogenesis research in many other organisms and could result in identifying potential broad-spectrum therapeutic targets to combat antibiotic-resistant bacteria, which represent a global crisis of the 21st century.

## Abstract (precis)

*Yersinia pestis* is the bacterial agent that causes the highly fatal disease plague. The organism represents a significant concern because of its potential use as a bioterror agent, beyond the several thousand naturally occurring human infection cases occurring globally each year. While there has been development of effective antibiotics, the narrow therapeutic window and challenges posed by the existence of antibiotic-resistant strains represent serious concerns. We sought to identify novel virulence factors that could potentially be incorporated into an attenuated vaccine platform or be targeted by novel therapeutics. We show here that a highly conserved quorum-sensing system, autoinducer-2, significantly affected the virulence of *Y. pestis* in a mouse model of pneumonic plague. We also identified steps in autoinducer-2 signaling which had confounded previous studies and demonstrated the potential for intervention in the virulence mechanism(s) of autoinducer-2. Our findings may have an impact on bacterial pathogenesis research in many other organisms and could result in identifying potential broad-spectrum therapeutic targets to combat antibiotic-resistant bacteria, which represent a global crisis of the 21st century.

## INTRODUCTION

Autoinducer-2 (AI-2), a quorum-sensing (QS) molecule found widely among Gram-positive and -negative bacteria, is associated with a diverse array of virulence mechanisms, ranging from secretion systems to biofilm formation in *in vitro* culture assays (1–7). Despite the linking of virulence mechanisms to AI-2 signaling, evidence of biological significance for these signaling pathways is limited in *in vivo* models (4, 7–9). Generally, the AI-2 signaling is characterized in a given organism by deleting the gene encoding the primary synthetic enzyme for the AI-2 substrate, LuxS, and observing changes in bacterial virulence phenotypes (10). During the course of our investigation into novel virulence factors of *Yersinia pestis*, the causative agent of plague, we reported a dramatic increase in attenuation of the Δ*lpp* Δ*msbB* Δ*rbsA* combinatorial deletion mutant in a stringent pneumonic plague mouse model (11). Our earlier studies showed that deletions of *lpp*, the gene encoding Braun lipoprotein (Lpp), and *msbB*, a gene encoding a lipopolysaccharide (LPS)-modifying acyltransferase (MsbB), attenuated a highly virulent *Y. pestis* strain, CO92 (12–14). While Lpp activates Toll-like receptor 2 (TLR-2) signaling, MsbB adds lauric acid to the lipid A moiety of LPS to modulate TLR-4 signaling (12). The additional deletion of *rbsA* (identified during our genome-wide, transposon-based, signature-tagged mutagenesis of *Y. pestis* CO92 [11]), encoding the ATP binding protein ribose ATP binding cassette (ABC) transporter, led to a further attenuation of the Δ*lpp ΔmsbB* mutant that was in excess of 10-fold (11). Investigation into the mechanism of the attenuation due to the deletion of *rbsA* within the *rbsBAC* operon showed that RbsA was necessary for efficient bacterial growth in a minimal medium limited to a ribose carbon source (11). While RbsA has ATPase activity, its coupling with RbsC, a bacterial membrane-associated protein, actively transports ribose that has been shuttled through the periplasm of the organism by high-affinity association with RbsB (15, 16).

In addition to the role in ribose utilization, orthologs of ribose transport proteins, such as RbsB in *Aggregatibacter actinomycetemcomitans*, efficiently interacted with AI-2 under physiologically relevant conditions (2, 9). The ribose transporter (Rbs), as well as the Lsr (LuxS-regulated) ABC transporter, are responsible for the uptake of the AI-2 QS signaling molecule into bacterial cells in many pathogenic bacteria which do not possess the dedicated two-component circuit of *Vibrio harveyi* (17). *V. harveyi* produces three autoinducers: AI-1 (3-hydroxybutanoyl homoserine lactone), CAI-1 [(*S*)-3-hydroxytridecan-4-one], and AI-2 [(2*S*,4*S*)-2-methyl-2,3,3,4-tetrahydroxytetrahydrofuranborate] (18), which are detected extracellularly by their cognate transmembrane receptors, namely, LuxN, CqsS, and LuxPQ, respectively (18). Signals through the autoinducer-sensing pathways are then transduced through shared components LuxU and LuxO and five small regulatory RNAs (sRNAs) to the master quorum-sensing regulator LuxR in *V. harveyi* (19, 20). An earlier study of AI-2 in an attenuated *Y. pestis* strain, KIM 1001 (with a deletion of the pigmentation locus [*pgm]* required for iron uptake), revealed significant expression changes in large sets of genes, as well as diminished oxidative damage resistance, when *luxS* was deleted from the Δ*pgm* mutant (7). The *luxS* gene encodes the AI-2 synthetic enzyme, while the *lsrK* gene encodes a kinase which phosphorylates AI-2, and the sequestered phospho-AI-2 then binds to the LsrR repressor to activate transcription of the *lsr* operon (21). However, deletion of the *luxS* gene from a fully virulent KIM5 strain of *Y. pestis* did not alter the 50% lethal dose (LD_50_) compared to that of the wild-type (WT) bacterium in a mouse model of bubonic plague (22). In this study, we demonstrated for the first time that the disruption of AI-2 transport from the extracellular milieu into *Y. pestis* CO92 due to the deletion of the *rbsA* and *lsrA* genes resulted in a significant reduction of virulence of the mutant in a mouse model of pneumonic plague. Furthermore, the deletion of the *luxS* or *lsrK* gene compromised the attenuated phenotype of the Δ*rbsA* Δ*lsrA* mutant, thus providing new insights into AI-2 signaling.

## RESULTS

### Deletion of *rbsA* and *lsrA* genes from *Y. pestis* CO92 disrupts autoinducer-2 signaling.

The initial finding we reported, that the deletion of the *rbsA* gene synergistically attenuated *Y. pestis* CO92 in association with deletions of *lpp* and *msbB* genes in a mouse model of pneumonic plague, led us to investigate mechanisms of attenuation beyond the impairment of ribose transport and utilization (11). Since orthologs of the Rbs operon are also associated with AI-2 transport, we examined the effect of combinatorial deletion of *lpp*, *msbB*, and *rbsA* on the levels of AI-2 in the culture supernatants of mutants versus the level in the supernatant of WT CO92. At temperatures of both 28°C (flea) and 37°C (human body), representing two lifestyles of *Y. pestis* (23), there were major aberrations in the patterns of AI-2 in the highly attenuated mutants (the Δ*lpp* Δ*msbB* and Δ*lpp* Δ*msbB ΔrbsA* mutants) compared to its occurrence in WT CO92 that were independent of the bacterial growth rates (data not shown). Interestingly, deletion of *rbsA* from the Δ*lpp* Δ*msbB* mutant had a potentiating effect on disrupting AI-2 patterns. This initial finding suggested that changes in AI-2 were potentiated with deletion of the *rbsA* gene but were inadequate to indicate a causal relationship, leading us to study the AI-2 signaling system in greater detail.

**FIG 1:**
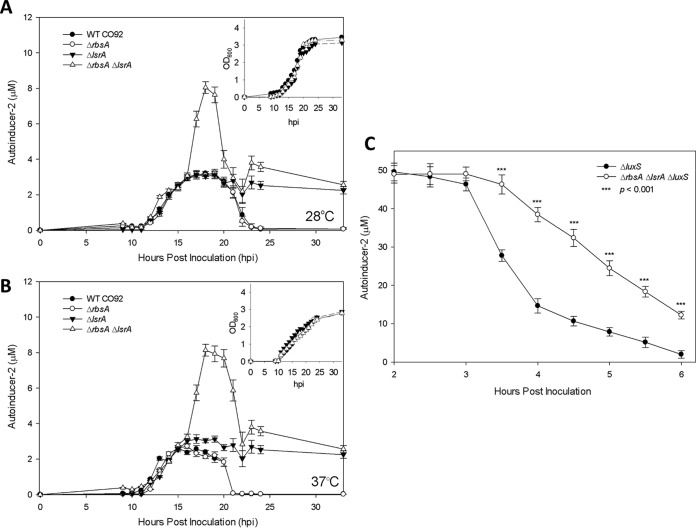
AI-2 levels in cell-free supernatants of WT *Y. pestis* CO92 and its various mutant strains. (A and B) Concentrations of AI-2 in culture supernatants during growth at 28°C (A) or 37°C (B) are shown, with absorbance of culture at 600 nm in insets. (C) AI-2 levels in cell-free supernatants following dosing of cultures with synthetic exogenous AI-2. Data are from three independent cultures per strain. Arithmetic means ± standard deviations from three independent experiments are shown. Statistical analysis was performed using pairwise *t* tests with multiple comparison correction.

Since the deletion of the *lpp* and *msbB* genes from *Y. pestis* could possibly affect the topology of the bacterial membrane and/or the expression of the stress response genes (23), we evaluated changes in the AI-2 levels of mutants with a deletion in the canonical AI-2 transport system both singly (*lsrA*) and in combination with *rbsA*. The *lsrA* gene, which encodes the ATPase component of the Lsr ABC transporter complex, was chosen because of the similarity of its function to that of RbsA, which is also an ATPase for the RbsBAC transporter complex. When the AI-2 levels were measured in serially diluted culture supernatants using the *V. harveyi* reporter strain BB170, the single deletion of *rbsA* resulted in no discernible effect on the AI-2 pattern when compared to that of WT CO92 at both temperatures, 28°C and 37°C (Fig. 1A and B). As previously reported (7), the *lsrA* deletion resulted in increases of AI-2 during stationary phase (after 22 h); however, when combined with the *rbsA* deletion, the Δ*rbsA* Δ*lsrA* mutant strain exhibited greater-than-two- to threefold increases in the levels of extracellular AI-2 during the mid- to late log phase of growth, as well as potentiation of the stationary-phase changes in the AI-2 levels observed with the Δ*lsrA* single mutant (Fig. 1A and B). The increases in extracellular AI-2 were observed both at 28°C and 37°C, with similar magnitudes of AI-2 increase.

The above-described changes in the extracellular levels of AI-2 could be the result of two competing processes: increased synthesis of AI-2 through the LuxS enzyme or decreased uptake of AI-2 from the extracellular milieu or both. To discriminate between these two possibilities, the synthesis of AI-2 was eliminated by the deletion of the *luxS* gene. The Δ*luxS* mutant was able to efficiently take up synthetic AI-2 from the culture medium during *in vitro* growth (Fig. 1C); however, the Δ*rbsA* Δ*lsrA* Δ*luxS* mutant was impaired in its ability to transport synthetic AI-2, with statistically significant delayed uptake and altered kinetics that led to a slow linear decrease in the concentration of AI-2 in the culture supernatant. The Δ*luxS* mutant with intact *rbs* and *lsr* operons exhibited a sigmoidal depletion of AI-2 that was initiated approximately 1 h prior to the Δ*rbsA* Δ*lsrA* Δ*luxS* mutant’s lower rate of AI-2 uptake (Fig. 1C).

### Changes in AI-2 signaling correlate to *in vitro* and *in vivo* attenuation of *Y. pestis* CO92.

**FIG 2:**
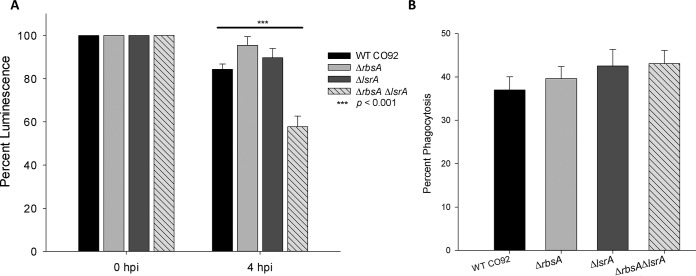
Intracellular survival of WT *Y. pestis* CO92 and its various mutant strains in macrophages. (A) The intracellular survival of WT *Y. pestis* and its various mutants in a RAW 264.7 murine macrophage cell line was evaluated at 0 and 4 h postinfection (hpi). Luminescent reporter strains from each background were utilized to evaluate real-time reporting of bacterial survival in macrophages. (B) Phagocytosis of bacteria was determined by comparing luminescence of infectious dose after culture to luminescence at 0 h. Data are from three independent experiments per strain. Arithmetic means ± standard deviations are shown. Statistical analysis was performed using one-way analysis of variance (ANOVA) with Tukey *post hoc* correction.

Following the confirmation of AI-2 aberrations, we determined whether there was any correlation between changes in AI-2 signaling and *in vitro* virulence as measured by intracellular survival (ICS) of the mutants in RAW 264.7 murine macrophages compared to the survival of WT CO92 (Fig. 2A). The ability to survive and replicate within macrophages and the recruitment of early immune effector cells during infection contribute to pathogenicity in *in vivo* models and, as such, are important measures of *Y. pestis* virulence (24, 25). We found that the single deletion of either of the transport protein-encoding genes (*rbsA* or *lsrA*) had a minimal effect on ICS, but when they were combined, the Δ*rbsA* Δ*lsrA* mutant was significantly less resistant to the macrophage intracellular environment (Fig. 2A). All of the mutants tested exhibited levels of phagocytosis similar to that of WT CO92 (Fig. 2B).

**FIG 3:**
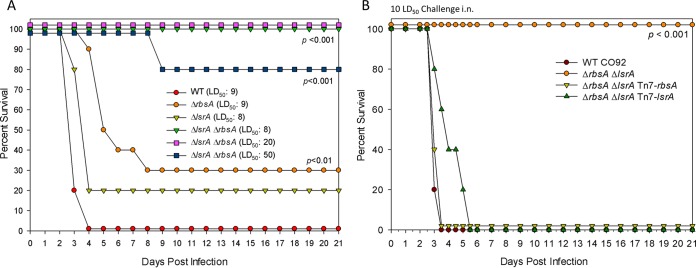
*In vivo* virulence of various *Y. pestis* mutants. (A and B) Survival of female Swiss-Webster mice (*n* = 5 to 10) in a pneumonic plague model after challenge with the stated dose (equivalent to WT LD_50_, where 1 LD_50_ is 500 CFU) of the *rbsA*, *lsrA*, or Δ*rbsA* Δ*lsrA* deletion mutant (A) or the Δ*rbsA* Δ*lsrA* double deletion strain complemented with the Tn*7* transposon (B). Data are representative of three independent experiments. Statistical analysis was performed using Kaplan-Meier survival curve analysis.

The data described thus far have been consistent with previous studies of AI-2 signaling in various pathogenic bacteria, showing changes in bacterial virulence related to *in vitro* assays. However, when we challenged mice in a pneumonic plague model to determine whether these changes in AI-2 signaling correlated with an alteration in *in vivo* virulence, our findings diverged from the previous literature (Fig. 3) (4, 8). The single gene deletion of either *rbsA* or *lsrA* showed only modest decreases in virulence in a mouse model; the survival of mice challenged with the Δ*rbsA* mutant reached statistical significance at 30% survival when a challenge dose equivalent to 9 LD_50_ of WT CO92 was used, while the survival of mice challenged with the Δ*lsrA* mutant was not significantly different (20% survival) from that of the WT CO92-challenged group of mice. The Δ*rbsA* Δ*lsrA* combinatorial deletion resulted in a significant decrease in the virulence of the mutant, with 80 to 100% of mice surviving a challenge dose of 8- to 50-LD_50_ equivalent of WT CO92 (Fig. 3A). These data for the Δ*rbsA* Δ*lsrA* mutant were comparable to our published results for virulence attenuation of the Δ*lpp* Δ*msbB* Δ*rbsA* strain, with 100% survival at a 50-LD_50_ equivalent of WT CO92 (11); the Δ*lpp* Δ*msbB* Δ*rbsA* strain also exhibited changes in AI-2 levels similar to those seen for the Δ*rbsA* Δ*lsrA* mutant (data not shown). The attenuated phenotype of the Δ*rbsA* Δ*lsrA* mutant could be complemented through a site-specific, single-copy, mini-Tn*7* transposon insertion of the native gene, along with the promoter, of either *rbsA* or *lsrA* (Fig. 3B). The significant decreases in virulence of the above-described mutants (the Δ*rbsA* Δ*lsrA* and Δ*lpp* Δ*msbB* Δ*rbsA* mutants) (11) that we observed in animals was unexpected, given the extensive literature indicating a negligible role for AI-2 in regulating *in vivo* virulence (4, 7, 8, 10, 26). As such, our results merited a more thorough investigation, and we decided to determine the additional role that *luxS* plays in virulence.

### Suppression of Δ*rbsA* Δ*lsrA* phenotype of decreased bacterial virulence *in vivo* by *luxS* deletion in *Y. pestis* CO92.

**FIG 4:**
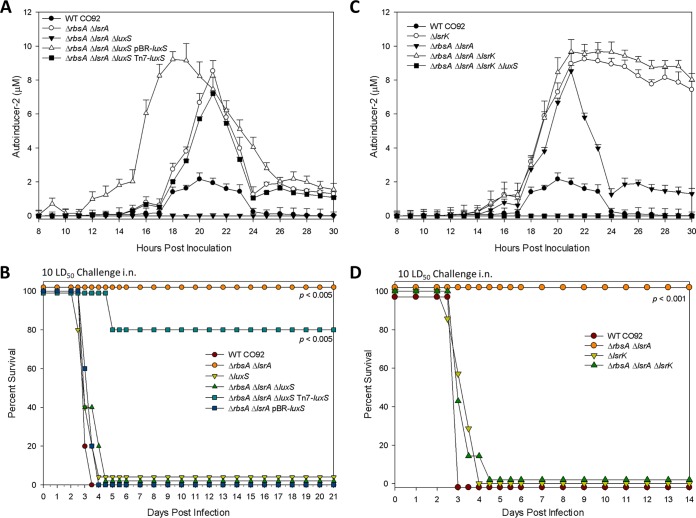
*In vivo* virulence and AI-2 levels in cell-free supernatants of Δ*luxS* and Δ*lsrK* mutants. (A and C) Concentrations of AI-2 in culture supernatants over a 30-h period of growth at 28°C. Error bars represent standard deviations from three independent experiments. (B and D) Survival of female Swiss-Webster mice (*n* = 5 to 10) in a pneumonic plague model after challenge with 10-LD_50_ equivalent of WT CO92, where 1 LD_50_ is 500 CFU. Data are representative of three independent experiments. Statistical analysis for animal studies was performed using Kaplan-Meier survival curve analysis.

To characterize the effect *luxS* deletion had on the AI-2 signaling pathway, we constructed single and combinatorial deletions of *luxS* in the background strains of WT CO92 and the Δ*rbsA* Δ*lsrA* mutant, in addition to developing various complemented and overexpressing strains. These strains were then characterized by determining the AI-2 levels in the culture supernatants over a period of bacterial growth. As expected, the deletion of *luxS* led to abrogation of AI-2 at all time points of bacterial growth tested (data not shown), which was replicated by the Δ*rbsA* Δ*lsrA* Δ*luxS* mutant (Fig. 4A). Interestingly, we found that the copy number of the *luxS* gene was critical in the context of AI-2 uptake during complementation of the Δ*rbsA* Δ*lsrA* Δ*luxS* mutant. *trans* complementation via a low-copy-number plasmid vector, pBR322, resulted in an early accumulation of AI-2 in the culture supernatant, with altered kinetics of depletion compared to that in the background Δ*rbsA* Δ*lsrA* mutant strain (Fig. 4A). Comparable AI-2 levels in the supernatants of the Δ*rbsA* Δ*lsrA* Δ*luxS* mutant and the Δ*rbsA* Δ*lsrA* background strain were only observed when complementation was achieved through *cis* complementation of a single copy of the *luxS* gene utilizing a Tn*7*-based transposon system (Fig. 4A).

We then evaluated the *luxS* deletion strains for their ability to cause disease in a pneumonic plague mouse model. We noted a significant trend not reported before in the literature (Fig. 4B). While the Δ*luxS* mutant was as virulent as the WT CO92, the deletion of *luxS* resulted in suppression of the attenuated phenotype of the Δ*rbsA* Δ*lsrA* background strain, thus attaining full virulence similar to that of WT CO92 (Fig. 4B). Interestingly, when bacterial CFU were evaluated in the lungs of mice at 24, 48, and 72 h and 7 days postinfection, it was noted that both the WT CO92 and the Δ*luxS* strain exhibited rapid growth up to 10^10^ CFU by 72 h postinfection (see Fig. S1A in the supplemental material). However, in the lungs of mice infected with the Δ*rbsA* Δ*lsrA* strain, bacterial counts persisted, albeit at much lower numbers and in the range of 10^2^ to 10^4^ CFU, out to 72 h postinfection before being cleared on day 7 postinfection (see Fig. S1A).

10.1128/mSphere.00342-16.1 Figure S1  Lung colonization and intracellular survival of WT *Y. pestis* CO92 and its Δ*luxS* and Δ*rbsA* Δ*lsrA* deletion mutants in mice (*n* = 4). (A) Bacterial lung counts were obtained from whole-lung homogenates harvested at 24, 48, or 72 h or 7 days postinfection with a 10-LD_50_ dose by the intranasal (i.n.) route in female Swiss-Webster mice. The horizontal line represents average counts from four animals. (B) Intracellular survival of Δ*luxS*, Δ*rbsA* Δ*lsrA*, or Δ*rbsA* Δ*lsrA* Δ*luxS* deletion mutant compared to that of WT CO92. The intracellular survival of WT CO92 and its various mutants in a RAW 264.7 murine macrophage cell line was evaluated at 0 and 4 h postinfection. Luminescent reporter strains from each background were utilized to evaluate real-time reporting of bacterial survival in macrophages. Statistical analysis was performed using one-way ANOVA with Tukey *post hoc* correction, and three independent experiments were performed. ***, *P* < 0.001; *, *P* < 0.01; hpi, hours postinfection; dpi, days postinfection. Download Figure S1, TIF file, 0.3 MB. Copyright © 2016 Fitts et al. 2016 Fitts et al. This content is distributed under the terms of the Creative Commons Attribution 4.0 International license.

We also found that the copy number of *luxS* modified the observed phenotype of the Δ*rbsA* Δ*lsrA* Δ*luxS* mutant. While the overexpression of *luxS* via the *trans* complementation strategy (with pBR322) resulted in a virulent phenotype (Fig. 4B), *cis* complementation of the Δ*rbsA* Δ*lsrA* Δ*luxS* mutant with *luxS* using the Tn*7-*based system led to an avirulent phenotype (80% survival) in a mouse model of pneumonic plague, possibly suggesting a dose-dependent effect of the *luxS* gene on *Y. pestis* virulence (Fig. 4B). Overall, our data obtained in the context of the Δ*rbsA* Δ*lsrA* mutant indicated that both the loss of *luxS*, as in the Δ*rbsA* Δ*lsrA* Δ*luxS* mutant, and the overexpression of *luxS*, as in the Δ*rbsA* Δ*lsrA*(pBR-*luxS*) mutant, led to virulent phenotypes *in vivo* (Fig. 4B). When we examined the ICS of these strains, we found a similar trend, where a single *luxS* deletion induced a phenotype similar to that of WT CO92 and suppression of attenuating characteristics in terms of ICS for the Δ*rbsA* Δ*lsrA* Δ*luxS* strain (see Fig. S1B in the supplemental material).

The paradigm of equating LuxS with AI-2 function has been questioned in the past, particularly due to multiple roles of LuxS beyond AI-2 substrate production (10). To verify whether the phenotype observed in *luxS* mutants was derived from its effects on AI-2, we generated a series of mutants that carried a deletion in the gene coding for the AI-2 kinase, *lsrK*. Prior literature has demonstrated that the deletion of *lsrK* results in an *Escherichia coli* strain that is both insensitive to AI-2 and unable to sequester AI-2, leading to a high concentration of it in the extracellular milieu that does not decrease over time (21, 27). We confirmed that this phenotype occurs in *Y. pestis* with a deletion of *lsrK*; interestingly, in the Δ*lsrK* and Δ*rbsA* Δ*lsrA* Δ*lsrK* mutants, the levels of AI-2 rise to concentrations similar to those seen for the Δ*rbsA* Δ*lsrA* mutant but then remain at these elevated levels through the stationary phase (Fig. 4C). As expected, the Δ*rbsA* Δ*lsrA* Δ*luxS* Δ*lsrK* quadruple mutant did not synthesize any AI-2. When *lsrK* mutants (Δ*lsrK* and Δ*rbsA* Δ*lsrA* Δ*lsrK* mutants) were tested for virulence in a pneumonic plague mouse model, they were universally lethal (Fig. 4D). In other words, the attenuated-virulence phenotype of the Δ*rbsA* Δ*lsrA* mutant was suppressed with the deletion of the *lsrK* gene, similar to the result for the *luxS* gene deletion mutant (Fig. 4B and D).

Thus far, we have shown a correlation between disruptions in AI-2 signaling due to aberrant transport of AI-2 within the bacterial cell and the attenuation of phenotypes in both *in vitro* and *in vivo* models of plague. Furthermore, modulation of AI-2 signaling due to the deletion of either the *luxS* gene or the downstream *lsrK* gene reverts the attenuated phenotype of the Δ*rbsA* Δ*lsrA* mutant to a phenotype similar to that of the WT bacterium.

### Transcriptomic profiles of *Y. pestis* CO92 strains in which AI-2 is perturbed.

**FIG 5:**
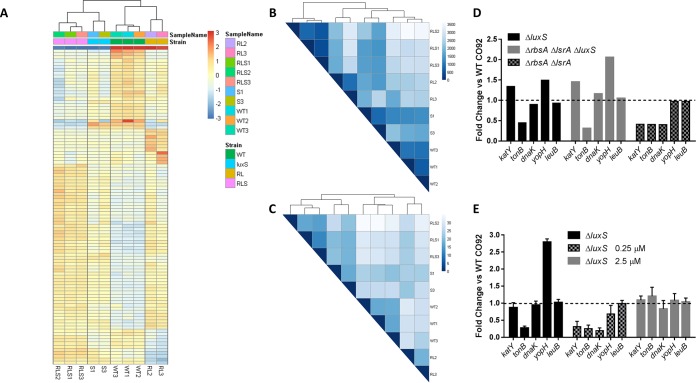
Analysis of AI-2 mutant transcriptomes. (A) A heat map was constructed against mean counts of the top 100 most variable genes across all samples, including WT CO92 (WT), Δ*rbsA* Δ*lsrA* (RL), Δ*luxS*, and Δ*rbsA* Δ*lsrA* Δ*luxS* (RLS) mutants. (B and C) Distance maps were created using Poisson (B) or Euclidean (C) distance against the transcriptome of each mutant examined. (D) Relative transcript levels from the RNA-seq data set were plotted for selected genes, with the level of each gene in each strain being compared to its level in WT CO92. (E) Relative expression levels of selected genes from qRT-PCR data were plotted for the indicated strains after growth with exogenous AI-2 added at the indicated concentrations. Standard deviations of the results from 3 independent experiments are shown.

To identify potential mechanism(s) of attenuation and further link the observed changes in AI-2 levels to the attenuated phenotypes of the mutants, we subjected each of the major mutant strains to high-throughput RNA sequencing (RNA-seq) analysis. RNA was isolated from the bacterial strains at peak AI-2 levels during the mid- to late exponential phase of growth, when the most significant aberrations in AI-2 signaling were observed (Fig. 1A and B). A heat map of the top 100 most-variable genes showed similar expression patterns within each strain and common expression patterns shared between strains in which AI-2 was perturbed, as well as isolated groupings unique to the attenuated Δ*rbsA* Δ*lsrA* strain (Fig. 5A). Distance mapping of the strains revealed a hierarchical grouping of the samples within their strains, exhibiting low variance between samples, as well as commonalities between the Δ*rbsA* Δ*lsrA* Δ*luxS* and Δ*luxS* strains (Fig. 5B and C).

Of the approximately 4,000 genes in the *Y. pestis* genome, 219 were differentially expressed between the Δ*rbsA* Δ*lsrA* strain and WT CO92 by more than twofold up or down at a significance level of *P*_adj_ of <0.05, with *P* adjusted for multiple comparisons as defined by DESeq2, 119 were differentially expressed between Δ*luxS* and WT CO92, 46 between the Δ*luxS* and Δ*rbsA* Δ*lsrA* Δ*luxS* strains, and 78 between the Δ*luxS* and Δ*rbsA* Δ*lsrA* strain (see Tables S1 and S2 in the supplemental material). From these data, we identified patterns of gene expression unique to the attenuation of the Δ*rbsA* Δ*lsrA* strain, changes that suppressed attenuation in the Δ*rbsA* Δ*lsrA* Δ*luxS* strain, and lastly, expression patterns common to both the Δ*rbsA* Δ*lsrA* and the Δ*luxS* mutant.

10.1128/mSphere.00342-16.5 Table S1  Significantly differently expressed genes in the Δ*rbsA* Δ*lsrA* strain versus WT CO92 and in the Δ*luxS* strain versus WT CO92. Download Table S1, PDF file, 1 MB. Copyright © 2016 Fitts et al. 2016 Fitts et al. This content is distributed under the terms of the Creative Commons Attribution 4.0 International license.

10.1128/mSphere.00342-16.6 Table S2  Significantly differently expressed genes in the Δ*luxS* strain versus the Δ*rbsA* Δ*lsrA* Δ*luxS* strain and in the Δ*luxS* strain versus the Δ*rbsA* Δ*lsrA* strain. Download Table S2, PDF file, 0.8 MB. Copyright © 2016 Fitts et al. 2016 Fitts et al. This content is distributed under the terms of the Creative Commons Attribution 4.0 International license.

Attenuating expression was identified by sorting for significant changes in the Δ*rbsA* Δ*lsrA* strain versus WT CO92 and excluding any significant changes common to the Δ*luxS* or Δ*rbsA* Δ*lsrA* Δ*luxS* strain and WT CO92 (see Table S3 in the supplemental material). Attenuating changes in expression (mean fold changes in expression are shown in parentheses below) included 249 genes with a *P*_adj_ of <0.1, comprising genes encoding ABC transporter families specific to arabinose (*araCFGH*) (−1.8 to −5.9) and galactose (*mglABC*) (−1.6 to −1.8), chaperone-encoding genes *dnaJK* (−2.4 to −2.5), *ibpAB* (−2.6), and *htpG* (−3.2), oxidative phosphorylation gene family *atpABEFGH* (1.6 to 2.2), anaerobic nitrogen metabolism gene family *napABC* (3.5 to 5.3), catalase gene *katY* (−2.42), and 44 genes encoding hypothetical proteins. Changes were also identified in metabolic pathway regulation genes, including a key regulator, *arcA* (1.52), as well as major phosphotransferase system (PTS) regulators *ptsG* (−2.14) and *ptsIH* (1.45 to 1.81).

10.1128/mSphere.00342-16.7 Table S3  Significantly differently expressed genes indicating an attenuated phenotype. Download Table S3, PDF file, 0.8 MB. Copyright © 2016 Fitts et al. 2016 Fitts et al. This content is distributed under the terms of the Creative Commons Attribution 4.0 International license.

An earlier study in which *luxS* was deleted in a *Y. pestis* Δ*pgm* strain showed decreased hydrogen peroxide resistance, with downregulation in the expression of the *katY* gene, compared to the hydrogen peroxide resistance of the parental strain (7). Since we also observed downregulation in the expression of the *katY* gene in association with attenuation, a hydrogen peroxide resistance assay was performed, and we found minimal decreases in the Δ*rbsA* or the Δ*lsrA* strains but a significant decrease in hydrogen peroxide resistance in the attenuated Δ*rbsA* Δ*lsrA* mutant strain (see Fig. S2A in the supplemental material), consistent with the decreased expression of *katY* (see Table S3).

10.1128/mSphere.00342-16.2 Figure S2  Functional assays of significant expression changes as determined by RNA-seq. (A) Resistance to killing by hydrogen peroxide. Resistance of indicated bacterial strains to killing by medium containing 0.3% H_2_O_2_ was evaluated through measurement of luminescence reporter in real-time. Measurements were taken 15 min after the addition of hydrogen peroxide. Statistical analysis was performed via one-way ANOVA with Tukey *post hoc* correction. (B) Growth of *Y. pestis* strains in carbon source-restricted minimal medium, measured by absorbance. *Y. pestis* strains were inoculated into a modified M9 medium, and samples were measured for absorbance at a wavelength of 600 nm every hour. Asterisks denote time points at which results for WT CO92 and the mutant were statistically significantly different at a *P* value of <0.001. Statistical analysis was performed using multiple *t* tests, with the Holm-Sidak method to correct for multiple comparisons. (C) Growth of *Y. pestis* strains in iron source-restricted minimal medium. *Y. pestis* strains were inoculated into a modified M9 medium, and samples were measured for absorbance at a wavelength of 600 nm every hour. Asterisks denote time points at which the results for WT CO92 and mutants were statistically significantly different at a *P* value of <0.001. Statistical analysis was performed using multiple *t* tests, with the Holm-Sidak method to correct for multiple comparisons. Data (arithmetic means ± standard deviations) are from three independent experiments. Download Figure S2, TIF file, 0.2 MB. Copyright © 2016 Fitts et al. 2016 Fitts et al. This content is distributed under the terms of the Creative Commons Attribution 4.0 International license.

The changes in metabolic regulator gene expression were also especially intriguing given the significant attenuation observed for the growth of *Y. pestis* in the macrophages in nutrient-limited environments, as well as in the *in vivo* mouse model. To determine whether changes in metabolic gene expression could be altering the growth pattern of the Δ*rbsA* Δ*lsrA* mutant in a restricted-nutrient environment, the mutant and the WT CO92 strain were grown in a modified defined medium based on M9 salts. The Δ*rbsA* Δ*lsrA* mutant exhibited delayed growth kinetics when glucose was used as the primary carbon source (see Fig. S2B in the supplemental material). There was an extended lag phase of growth for the mutant, although it reached a final optical density (OD) equivalent to that of WT CO92.

Phenotype-suppressing gene expression was identified by sorting for unique changes between the Δ*rbsA* Δ*lsrA* mutant and WT CO92 that were also not evident between the Δ*rbsA* Δ*lsrA* Δ*luxS* mutant and WT CO92 (see Table S4 in the supplemental material). Through this analysis, 220 genes with a *P*_adj_ of <0.1 were selected, which included a large segment of the genes encoding the type III secretion system (T3SS), including structural genes *yscABCDGLOPRSTUVXY* (1.3 to 2.6) and effector protein genes *yopBDHJMQRT* (1.4 to 2.9); all of these genes were upregulated, indicating globally increased expression of the T3SS genes.

10.1128/mSphere.00342-16.8 Table S4  Significantly differently expressed genes indicating suppression of attenuation. Download Table S4, PDF file, 0.8 MB. Copyright © 2016 Fitts et al. 2016 Fitts et al. This content is distributed under the terms of the Creative Commons Attribution 4.0 International license.

The T3SS in *Y. pestis* has been extensively characterized as an essential virulence system with functions ranging from targeted cell lysis to immune evasion (28). We confirmed changes in the expression profiles of T3SS effectors, i.e., *Yersinia* outer protein E (YopE) and the structural low calcium response V antigen (LcrV), by Western blot analysis in *luxS-*associated and Δ*rbsA* Δ*lsrA* mutants, as well as WT CO92. Both of these proteins were secreted at higher levels in Δ*luxS* and Δ*rbsA* Δ*lsrA* Δ*luxS* mutants than in either WT CO92 or the Δ*rbsA* Δ*lsrA* mutant under inducing growth conditions *in vitro*, i.e., low calcium and 37°C (see Fig. S3A and B in the supplemental material) (28). Other virulence factor-encoding genes characterized, such as *ail*, the attachment and invasion locus, and *pla*, encoding plasminogen activator protease, were not significantly differentially expressed in any of the strains examined compared to their expression in WT CO92. We examined the levels of Pla by Western blot analysis (see Fig. S4B) and evaluated Pla protease activity (see Fig. S4A), and we found no significant difference among all mutants examined compared to the Pla level and activity of WT CO92.

10.1128/mSphere.00342-16.3 Figure S3  Type III secretion system function. (A) Western blots of anti-LcrV and anti-YopE antibodies from concentrated culture supernatants normalized against total protein visualized on the blot. (B and C) Densities of anti-YopE (B) and anti-LcrV (C) antibodies were measured and plotted. Western blot image is representative of three independent experiments. Arithmetic means ± standard deviations are shown. Download Figure S3, TIF file, 0.8 MB. Copyright © 2016 Fitts et al. 2016 Fitts et al. This content is distributed under the terms of the Creative Commons Attribution 4.0 International license.

10.1128/mSphere.00342-16.4 Figure S4  Pla protease activities and levels. (A) The activity of Pla protease was measured for several strains using a fluorescent reporter assay. (B) The protein levels of Pla were determined by immunoblotting using polyclonal anti-Pla antibodies and with loading controlled by total protein visualization using stain-free technology (Bio-Rad). Data are from three independent cultures per strain. Arithmetic means ± standard deviations are shown. Download Figure S4, TIF file, 0.3 MB. Copyright © 2016 Fitts et al. 2016 Fitts et al. This content is distributed under the terms of the Creative Commons Attribution 4.0 International license.

Finally, we identified changes in gene expression that were common to the Δ*rbsA* Δ*lsrA* and the Δ*luxS* mutant versus WT CO92 (see Table S5 in the supplemental material). This analysis identified 348 differentially expressed genes with a *P*_adj_ of <0.1 and included several iron transport gene families, as well as AI-1 quorum-sensing components. There were significant changes in the expression of iron transport-related genes. Inorganic chelated iron transport genes *yfeABCDE* (2.15 to 3.75) were upregulated, while the organic iron transport gene *tonB* (−2.8) and siderophore yersiniabactin synthesis genes *irp1* to *-8* (−1.83 to −3.29) were both uniformly repressed. Iron transport is essential for the full virulence of *Y. pestis*, as has been demonstrated through deletions of the *pgm* locus, which includes the yersiniabactin synthetic family, the *irp* genes (29).

10.1128/mSphere.00342-16.9 Table S5  Significantly differently expressed genes in response to autoinducer-2 dysregulation. Download Table S5, PDF file, 0.9 MB. Copyright © 2016 Fitts et al. 2016 Fitts et al. This content is distributed under the terms of the Creative Commons Attribution 4.0 International license.

To confirm the physiologic effects of these expression changes, we evaluated the growth of mutants in restricted-nutrient medium with a sole iron source of nonchelated inorganic iron, FeSO_4_. Restriction of the iron source resulted in delayed growth of the Δ*rbsA* Δ*lsrA* Δ*luxS* and Δ*luxS* mutants compared to the growth of WT CO92, as well as a lower final bacterial density, indicating a functional consequence of expression changes (see Fig. S2C in the supplemental material). In addition to the alterations in iron uptake mechanisms that were observed across AI-2-perturbed strains, we also observed uniform upregulation of AI-1 system component-encoding genes *ypeIR* (4.14 to 4.35) and *yspI* (2.02), including synthetic genes for both of the *acyl*-homoserine lactones used by the AI-1 system, as well as the downstream receptor (see Table S5).

### AI-2 concentration dependence of *Y. pestis* CO92 virulence.

**FIG 6:**
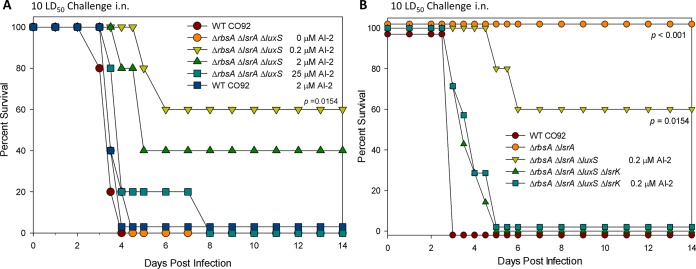
*In vivo* complementation of the Δ*rbsA* Δ*lsrA* Δ*luxS* mutant with exogenous synthetic AI-2. (A) Survival of female Swiss-Webster mice (*n* = 5) in a pneumonic plague model after challenge with 10-LD_50_ equivalent of WT CO92, where 1 LD_50_ is 500 CFU. Mice were dosed with the indicated concentrations of synthetic AI-2 in PBS via the intranasal (i.n.) route at the time of infection and at 24 h and 48 h postinfection. Data are representative of three independent experiments. Statistical analysis was performed using Kaplan-Meier survival curve analysis.

The transportation defect we identified in the Δ*rbsA* Δ*lsrA* mutant appeared to decrease early uptake of AI-2 into the bacteria, thus decreasing the amount of AI-2 available intracellularly. While the lack of AI-2 resulted in virulence comparable to that of WT CO92 *in vivo*, as was seen with *luxS* mutants, we were interested in the potential role of intermediate levels of AI-2, as might be occurring with the Δ*rbsA* Δ*lsrA* mutant strain. As has been reported previously by Rickard et al., responses to AI-2 can be dose dependent and bacteria may be sensitive to much lower levels of AI-2 than previously appreciated (30). Thus, we chemically rescued the Δ*luxS* strain with various concentrations of exogenous AI-2, ranging from 0 to the physiological level of AI-2, which we defined as the maximum concentration of AI-2 (2.5 µM) observed in the WT CO92 strain (Fig. 6). When we used quantitative real-time PCR (qRT-PCR) to analyze the expression of several key genes identified by RNA-seq (Fig. 5D), we found that the *luxS* deletion mutant with subphysiologic levels of AI-2 (0.25 µM) had decreased expression of *katY*, *tonB*, *dnaK*, and *yopH* (Fig. 5E), resembling the expression pattern of the Δ*rbsA* Δ*lsrA* mutant (Fig. 5D). Lack of AI-2 supplementation resulted in an expression profile with trends similar to those observed for the *luxS* mutant using RNA-seq (Fig. 5D and E), confirming the validity of those measures. Physiologic levels of AI-2 (2.5 µM) resulted in a gene expression profile in the *luxS* mutant that was similar to that of WT CO92 based on qRT-PCR (Fig. 5E).

Finally, we attempted to rescue phenotypes *in vivo* with exogenous AI-2. In the mouse pneumonic plague model, we partially rescued the Δ*rbsA* Δ*lsrA* Δ*luxS* mutant with exogenous AI-2 to an attenuated phenotype at doses calculated to achieve concentrations of 0.2 and 2 µM in the lungs of mice (Fig. 6A and B). Mice that were infected with the WT CO92 strain and received equivalent doses of AI-2 showed no difference in virulence (Fig. 6A), suggesting that the change in virulence is specific to the Δ*rbsA* Δ*lsrA* Δ*luxS* strain and due to the availability of exogenous AI-2. There was significantly greater attenuation in a group of mice infected with the Δ*rbsA* Δ*lsrA* Δ*luxS* mutant and receiving 0.2 µM of AI-2 than in animals receiving no exogenous AI-2 (*P* = 0.0154). Increasing the exogenous concentration of AI-2 to 25 µM did not provide any protection to mice when challenged with the Δ*rbsA* Δ*lsrA* Δ*luxS* mutant (Fig. 6A). Additionally, the Δ*rbsA* Δ*lsrA* Δ*luxS* Δ*lsrK* mutant strain, which was insensitive to AI-2, could not be rescued by the addition of AI-2 and was fully virulent regardless of the dose of exogenous AI-2 provided (Fig. 6B).

## DISCUSSION

The autoinducer-2 system has been proposed to be an interspecies metabolic-status signaling mechanism in bacteria, allowing adaptive regulation in response to environmental conditions. AI-2 controls a diverse array of traits in both nonpathogenic and pathogenic bacteria. In a Δ*rbsA* Δ*lsrA* mutant strain, we showed that aberrations in the AI-2 signaling mechanism resulted in a drastic reduction in virulence, a more-than-50-fold change as determined by LD_50_, compared to the virulence of the WT CO92 strain. This contrasts greatly with previous reports of AI-2 regulation both in *Y. pestis* and in other pathogenic bacteria (7, 10). We also demonstrated a basis for the reported differences observed in the context of AI-2 in virulence *in vivo* through deletion of the *luxS* gene and observation of its suppression of Δ*rbsA* Δ*lsrA* attenuation.

As has often been discussed in the literature, the role of LuxS in the cycling of homocysteine could have significant effects on the metabolic activity of bacteria. Furthermore, the loss of AI-2 production due to *luxS* deletion or, alternately, an endogenous AI-2 signal propagation that is distinct from exogenous signal propagation could play an important role in bacterial virulence (31). However, we showed that deletion of the *lsrK* gene from WT CO92 also resulted in a similar suppressive phenotype in a background of mutant (Δ*rbsA* Δ*lsrA*) attenuation, like the *luxS* deletion. LsrK phosphorylates AI-2, which binds LsrR, a repressor, inactivating it to trigger expression of the *lsr* operon (21, 27). These results suggest that the suppression of bacterial attenuation is due to the lack of AI-2 activity rather than the pleiotropic consequences of *luxS* deletion. Interestingly, the suppressive phenotype seems to result not from reversion to WT CO92 gene expression patterns but, rather, from upregulation of the T3SS of *Y. pestis*. This is not an isolated phenomenon either, as the upregulation of T3SS observed in this study due to the lack of AI-2 activity parallels similar secretion phenotypes in both *Aeromonas* and *Salmonella* Δ*luxS* strains (4, 32). The dose dependence of gene expression profiles, as well as rescue of the Δ*rbsA* Δ*lsrA* Δ*luxS* mutant from lethal effects in an *in vivo* model with subphysiological concentrations of AI-2, reveal a new mechanism of AI-2 signaling. Our data support a dose-dependent signaling model in which the response to AI-2 is stratified into three categories: zero activity, low activity, and high activity. Much of the previous literature has focused on the zero- and high-activity categories of AI-2, and, based upon this model, may be missing the significant intermediate category in which we observed the greatest disruption in virulence. We suggest that, in light of our results, AI-2 signaling may require reevaluation in other well-studied bacterial pathogens to characterize phenotypes derived from subphysiologic concentrations of AI-2 rather than complete absence of AI-2.

The results obtained through RNA-seq contain an incredible density of data that may point to mechanisms of attenuation and the role of AI-2 in bacterial systems. The metabolic regulation observed with AI-2 perturbation, especially the PTS system, indicates a strong role for AI-2 in the adaptive response to different environmental niches. The PTS system regulates preferred sugar uptake and depends on the flux of sugars to balance the phosphorylation state of two major regulatory kinases, PtsIH, that influence both the transcription and function of non-PTS transporters. The diminished expression of *ptsG* could influence the phosphorylation states of these kinases, in addition to the expression changes identified for *ptsIH*, allowing further dysregulation. Previous studies have indicated that PtsI is essential for the uptake of AI-2 via a regulatory function (33), and thus, the reciprocal changes in the expression of PTS regulators suggest a complex and strictly controlled dynamic that utilizes AI-2 as a powerful environmental signal. Taken together, the attenuated virulence phenotype of a *Y. pestis* CO92 mutant (e.g., the Δ*rbsA* Δ*lsrA* mutant) that has a diminished ability to transport AI-2 and the modulation of gene expression suggest a decoupling of metabolic status from regulatory control, resulting in a maladaptive metabolic and stress response profile.

Finally, the high conservation of AI-2 transport mechanisms and signaling pathways in microbes presents a significant opportunity for small-molecule intervention (34). Current inhibitors of AI-2 have unknown activity in *in vivo* models of disease. The paucity of *in vivo* data, in conjunction with the lack of attenuation previously observed for *luxS* deletion strains of several pathogenic bacteria, have prevented further research into the utility of AI-2 inhibitors. The inhibitors characterized thus far and the development of potential drug targets in the RbsBAC and LsrABCD families of proteins represent an untapped resource in the fight against antibacterial resistance.

## MATERIALS AND METHODS

### Bacterial strains, plasmids, and cell culture.

The bacterial strains used in this study are described in Table 1. *Y. pestis* strains were cultured overnight at 28°C, unless specifically noted otherwise in the figure legends, with shaking at 180 rpm in heart infusion broth (HIB) (Difco; Voigt Global Distribution, Inc., Lawrence, KS) or grown for 48 h on 5% sheep blood agar (SBA) (Teknova, Hollister, CA) or HIB agar plates. As appropriate, the organisms were cultivated in the presence of antibiotics ampicillin, kanamycin, and polymyxin B at concentrations of 100, 50, and 35 µg/ml, respectively. All of the experiments with *Y. pestis* were performed in the Centers for Disease Control and Prevention (CDC)-approved select agent laboratory in the Galveston National Laboratory (GNL), University of Texas Medical Branch (UTMB).

**TABLE 1:** Bacterial strains used in this study

Genotype	Description	Origin
WT CO92	Virulent *Y. pestis* biovar orientalis strain isolated in 1992 from a fatal human pneumonic plague case and naturally resistant to polymyxin B	CDC
Δ*rbsA*	*rbsA* deletion mutant of *Y. pestis* CO92	11
Δ*lpp* Δ*rbsA*	*lpp rbsA* double deletion mutant of *Y. pestis* CO92	11
*Δlpp* Δ*msbB* Δ*rbsA*	*lpp msbB rbsA* triple deletion mutant of *Y. pestis* CO92	11
Δ*lsrA*	*lsrA* deletion mutant of *Y. pestis* CO92	This study
*ΔrbsA* Δ*lsrA*	*rbsA lsrA* double deletion mutant of *Y. pestis* CO92	This study
Δ*luxS*	*luxS* deletion mutant of *Y. pestis* CO92	This study
*ΔrbsA* Δ*luxS*	*rbsA luxS* double deletion mutant of *Y. pestis* CO92	This study
Δ*lsrA* Δ*luxS*	*lsrA luxS* double deletion mutant of *Y. pestis* CO92	This study
*ΔrbsA* Δ*lsrA* Δ*luxS*	*rbsA lsrA luxS* triple deletion mutant of *Y. pestis* CO92	This study
Δ*rbsA* Δ*lsrA*(pBR-*luxS*)	*rbsA lsrA* double deletion mutant of *Y. pestis* CO92 transformed with pBR-*luxS* (Tc^s^)	This study
*ΔrbsA* Δ*lsrA* Δ*luxS*(pBR-*luxS*)	*rbsA lsrA luxS* triple deletion mutant of *Y. pestis* CO92 transformed with pBR-*luxS* (Tc^s^)	This study
Δ*rbsA* Δ*lsrA* Δ*luxS*::Tn*7*-*luxS*	*rbsA lsrA luxS* triple deletion mutant of *Y. pestis* CO92 integrated with a Tn*7* cassette carrying the native promoter and gene for *luxS*	This study
*ΔrbsA* Δ*lsrA*::Tn*7*-*rbsA*	*rbsA lsrA* double deletion mutant of *Y. pestis* CO92 integrated with a Tn*7* cassette carrying the native promoter and gene for *rbsA*	This study
Δ*rbsA* Δ*lsrA*::Tn*7*-*lsrA*	*rbsA lsrA* double deletion mutant of *Y. pestis* CO92 integrated with a Tn*7* cassette carrying the native promoter and gene for *lsrA*	This study
WT CO92::Tn*7*-*lux*	*Y. pestis* CO92 integrated with a Tn*7* cassette carrying the luciferase operon *luxCDABE* (*lux*) (Km^s^)	This study
*ΔrbsA*::*Tn7-lux*	*rbsA* deletion mutant of *Y. pestis* CO92 integrated with a Tn*7* cassette carrying the luciferase operon *luxCDABE* (*lux*) (Km^s^)	This study
*Δlpp* Δ*rbsA*::*Tn7-lux*	*lpp rbsA* double deletion mutant of *Y. pestis* CO92 integrated with a Tn*7* cassette carrying the luciferase operon *luxCDABE* (*lux*) (Km^s^)	This study
Δ*lpp* Δ*msbB* Δ*rbsA*::*Tn7-lux*	*lpp msbB rbsA* triple deletion mutant of *Y. pestis* CO92 integrated with a Tn*7* cassette carrying the luciferase operon *luxCDABE* (*lux*) (Km^s^)	This study
*ΔlsrA*::*Tn7-lux*	*lsrA* deletion mutant of *Y. pestis* CO92 integrated with a Tn*7* cassette carrying the luciferase operon *luxCDABE* (*lux*) (Km^s^)	This study
Δ*rbsA* Δ*lsrA*::*Tn7-lux*	*rbsA lsrA* double deletion mutant of *Y. pestis* CO92 integrated with a Tn*7* cassette carrying the luciferase operon luxCDABE (*lux*) (Km^s^)	This study
*ΔluxS*::*Tn7-lux*	*luxS* deletion mutant of *Y. pestis* CO92 integrated with a Tn*7* cassette carrying the luciferase operon *luxCDABE* (*lux*) (Km^s^)	This study
Δ*rbsA* Δ*luxS*::*Tn7-lux*	*rbsA luxS* double deletion mutant of *Y. pestis* CO92 integrated with a Tn*7* cassette carrying the luciferase operon *luxCDABE* (*lux*) (Km^s^)	This study
*ΔlsrA* Δ*luxS*::*Tn7-lux*	*lsrA luxS* double deletion mutant of *Y. pestis* CO92 integrated with a Tn*7* cassette carrying the luciferase operon *luxCDABE* (*lux*) (Km^s^)	This study
Δ*rbsA* Δ*lsrA* Δ*luxS*::*Tn7-lux*	*rbsA lsrA luxS* triple deletion mutant of *Y. pestis* CO92 integrated with a Tn*7* cassette carrying the luciferase operon *luxCDABE* (*lux*) (Km^s^)	This study
Δ*lsrK*	*lsrK* deletion mutant of *Y. pestis* CO92	This study
*ΔrbsA* Δ*lsrA* Δ*lsrK*	*rbsA lsrA lsrK* triple deletion mutant of *Y. pestis* CO92	This study
*ΔrbsA* Δ*lsrA* Δ*luxS ΔlsrK*	*rbsA lsrA luxS lsrK* quadruple deletion mutant of *Y. pestis* CO92	This study

RAW 264.7 murine macrophage cell lines (ATCC, Manassas, VA) were maintained in Dulbecco modified Eagle medium (DMEM) with 10% fetal bovine serum supplemented with 1% l-glutamine (Cellgro, Manassas, VA) and 1% penicillin-streptomycin (Invitrogen, Carlsbad, CA) at 37°C with 5% CO_2_.

### Construction of flippase expression plasmid.

An easily curable plasmid for the expression of flippase recombinase, pEF01, was constructed on a pCP20 backbone that incorporated a levansucrase (*sacB*) gene derived from pDMS197 and lacked the chloramphenicol resistance cassette. The construction of the plasmid was accomplished using In-Fusion cloning (Clontech Laboratories, Inc., Mountain View, CA) with double-stranded DNA (dsDNA) fragments generated by PCR with primers P1 to P6 (Table 2).

**TABLE 2:** Description of primers used in study

Primer	Description	Sequence (5′→3′)
P1	FLP, ampicillin, forward	CGCCTGTAGTGCCATTTACC
P2	FLP, ampicillin, reverse	CATTACGCTGACTTGACGGG
P3	FLP, forward	CCCGTCAAGTCAGCGTAATG
P4	FLP, reverse	GGTAGCGTTGCCAATGATGT
P5	SacB, FLP, forward	AATGGCACTACAGGCGTGGGAATTCTGATCCTTTTTAACCCATCAC
P6	SacB, FLP, reverse	TCATTGGCAACGCTACCGCCATTTGCCTGCTTTTATATAGT
P7	λ Red, LsrA, forward	ATTTGTTCAGTCCCGTCAGTCAACATTGAGGGAGCGGAGGCAACATGCAAGTGTAGGCTGGAGCTGCTTC
P8	λ Red, LsrA, reverse	CCGGTTATTTTGGATGAATTTCAACATGTTGCCTCCGACGCACCATGTTCCGGGGATCCGTCGACC
P9	λ Red, LuxS, forward	TTAGAAAAATATGACTTTTTTATGAGGAGGTAACTAAATGCCATTATTGGGTGTAGGCTGGAGCTGCTTC
P10	λ Red, LuxS, reverse	CGCCTTTTATCATTCTCCTGCCTACTGATACTGAGCACTAAATATGCAATATTCCGGGGATCCGTCGACC
P11	LsrA, Tn*7*, forward	CCAACACTCGAGAGGGCAAATAGGGTGAGAATG
P12	LsrA, Tn*7*, reverse	TCCTTCGAATTCAGCCACTGCGTAATGAATGTTT
P13	LsrA, reverse, sequencing	ATCTATCACCCCAGACTGCC
P14	LsrA, forward, sequencing	CCATCACGCCGTTCATTGAA
P15	LuxS, pBR322/Tn*7*, forward	TCCTTCGAATTCGCTTTGAAGAGTATTTAGCGCT
P16	LuxS, Tn*7*, reverse	CCAACAGGTACCAGCTTTACTGAACCCCCAGCC
P17	LuxS, pBR322, reverse	CCAACAGTCGACAAAGCTTTACTGAACCCCCAGCC
P18	LuxS, forward, sequencing	CAGTTATCTGCAGAGCGCGA
P19	LuxS, reverse, sequencing	GACGCTTTAATCAGCGCCTT
P20	λ Red, LsrK, forward	AGGGTATTCAAGAGGAGCGCGCAATGAGTCAACTCGATACGACTACCCCAGTGTAGGCTGGAGCTGCTTC
P21	λ Red, LsrK, reverse	AATGAAGATAATCCATTTTAGAGGCAAGGAGCCTTCCAAAGAGACGTCGTTTCCGGGGATCCGTCGACCT
P22	LsrK, reverse, sequencing	CGCCTTTAATCCTGCATGCT
P23	LsrK, forward, sequencing	CAGATATTGCGGTCGTTGGG

### Construction of in-frame deletion mutants.

To construct in-frame deletion mutants of *Y. pestis* CO92, the λ phage recombination system was used (35). Initially, the WT CO92 strain was transformed with plasmid pKD46 and grown in the presence of 1 mM l-arabinose to induce the expression of the λ phage recombination system. The above-mentioned *Y. pestis* culture was processed for the preparation of electroporation-competent cells (35, 36). The latter were then transformed with 0.5 to 1.0 µg of the linear dsDNA constructs carrying the kanamycin resistance (Km^r^) gene cassette, which was immediately flanked by the bacterial flippase recognition target (FRT) sequence, followed on either side by 50 bp of DNA sequences homologous to the 5′ and 3′ ends of the gene to be deleted from WT CO92. Plasmid pKD46 was cured from the mutants that had successful Km^r^ gene cassette integration at the correct location by growing the bacteria at 37°C. The latter mutants were transformed with plasmid pEF01 to excise the Km^r^ gene cassette. Eventually, plasmid pEF01 was also cured from the kanamycin-sensitive (Km^s^) clones by growing them at 37°C, followed by selection in a medium containing 5% sucrose (37). To confirm the in-frame deletion, mutants showing sensitivity to kanamycin and ampicillin were tested by PCR using appropriate primer pairs **(**Table 2**)** and sequencing of the PCR products.

### Growth curve and AI-2 determination of mutants.

To determine the AI-2 secretion profile, overnight cultures of bacteria were inoculated in HIB medium at a dilution of 1:1,000, and then aliquots of culture were taken at hourly intervals. The culture medium was centrifuged briefly and then filtered through 0.1-μm microcentrifuge filters (Corning Inc, Corning, NY) before being stored at −80°C prior to analysis. Analysis was performed as previously described (38); in brief, *V. harveyi* BB170 (ATCC), which is unable to synthesize AI-2, was inoculated into AB medium, incubated overnight at 30°C, and then diluted 1:5,000 in fresh AB medium (38). Freshly diluted *V. harveyi* BB170 was then mixed 9:1 with filtered culture supernatants of *Y. pestis* strains, and the mixture incubated for 5 h at 30°C. Samples were then analyzed for bioluminescence, and AI-2 concentrations determined by a standard curve obtained with synthetic AI-2 (Omm Scientific, Dallas, TX).

### Development of luminescent reporter strains.

Electrocompetent cells of *Y. pestis* strains were prepared and electroporated with pTNS2 and pUC18r6kT mini-Tn*7*T::*lux*-FRT-kan (39) and selected by kanamycin resistance and luminescence. Following isolation, strains were electroporated with pEF01 to remove the resistance cassette. Kanamycin-sensitive mutants were grown at 37°C and selected for on medium containing 5% sucrose for removal of pEF01. The insertion of the *lux* (luciferase) operon at the *att*Tn*7* region and appropriate removal of the kanamycin cassette were confirmed by PCR and Sanger sequencing. The luminescence intensity of each strain was determined by serial dilution and relative luminescence unit (RLU) measurement (Spectramax M5e; Molecular Devices, Sunnyvale, CA).

### Intracellular survival of *Y. pestis* CO92 strains in RAW 264.7 murine macrophages.

Intracellular survival of *Y. pestis* strains was determined as previously described (40). In brief, luminescent *Y. pestis* strains were grown in HIB overnight to saturation at 28°C. RAW 264.7 macrophages were seeded in 96-well plates at a concentration of 2 × 10^4^ cells/well for confluence. Plates were then infected with *Y. pestis* CO92 *lux* or the various mutant strains, also with *lux*, at a multiplicity of infection (MOI) of 250 in DMEM, centrifuged, and incubated at 37°C and 5% CO_2_ for 60 min. Infected macrophages were then washed with phosphate-buffered saline (PBS), treated with gentamicin, washed again with PBS, and maintained in DMEM as described above. At 0 and 4 h, luminescence was measured in a SpectraMax M5e microplate reader.

### *Y. pestis* CO92 pneumonic plague mouse model.

All of the animal studies with *Y. pestis* were performed in an animal biosafety level 3 (ABSL-3) facility under an approved Institutional Animal Care and Use Committee (IACUC) protocol (UTMB). Six- to 8-week-old female Swiss Webster mice (17 to 20 g), purchased from Taconic Laboratories (Germantown, NY), were anesthetized by the intraperitoneal route with a mixture of ketamine and xylazine and subsequently challenged intranasally with the indicated (as shown in the figures) LD_50_ doses (1 LD_50_ = 500 CFU) as described for WT *Y. pestis* CO92 (41). Mice were assessed for morbidity and/or mortality, as well as clinical symptoms, for the duration of each experiment (up to 21 days postinfection).

For the AI-2 complementation study, mice were anesthetized by isoflurane and dosed intranasally at the time of infection and at 24 h and 48 h postinfection with 20 μl of PBS with added synthetic AI-2 calculated to result in a 0, 0.2, 2, or 25 μM concentration of AI-2 in the lung volume of a 6- to 8-week-old female Swiss Webster mouse (~500 μl).

### AI-2 uptake by *Y. pestis.*

AI-2 uptake was determined as previously described (33). In brief, strains were grown to saturation overnight in HIB at 37°C. Bacteria were washed twice with PBS and diluted 1:100 in fresh HIB supplemented with 50 μM AI-2. Culture aliquots were sampled and assayed for AI-2 levels as described above.

### Hydrogen peroxide resistance of *Y. pestis* strains.

Luminescent reporter *Y. pestis* strains were cultured as described for AI-2 analysis, but bacteria were harvested at the time of maximal AI-2 production, washed twice in PBS, and resuspended at an optical density at 600 nm (OD_600_) of 1 in HIB supplemented with 0.3% H_2_O_2_ (Thermo Fisher Scientific, Waltham, MA). Luminescence was measured in a SpectraMax M5e microplate reader.

### Growth curve in modified minimal medium.

Overnight cultures of various *Y. pestis* strains were washed in PBS and then normalized by OD_600_. Flasks containing 20 ml of modified M9 medium (1× M9 salts [22 mM KH_2_PO_4_, 33.7 mM Na_2_HPO_4_, 8.55 mM NaCl, 9.35 mM NH_4_Cl], 1 mM MgSO_4_, 2.5 mM CaCl_2_, 0.001 mg/ml FeSO_4_, 0.0001% thiamine, 0.1% Casamino acids; all chemicals obtained from Sigma-Aldrich, St. Louis, MO) were supplemented with 0.4% glucose, inoculated with approximately 1 × 10^7^ CFU of various bacterial strains, and incubated at 37°C with shaking at 180 rpm. Samples were taken every hour, and absorbance measured at OD_600_.

### RNA-seq and expression analysis.

Cultures were grown as described for AI-2 growth curve analysis, and RNA was isolated at the time of peak AI-2 levels by using TRIzol (Thermo Fisher Scientific, Waltham, MA) and extracting with chloroform and ethanol. Total RNA was purified and DNase treated using the Quick-RNA kit (Zymo Research, Irvine, CA), followed by mRNA enrichment using MicrobeExpress (Ambion, Thermo Fisher Scientific, Waltham, MA).

### Library construction and sequencing.

RNA (1 to 3 µg) was fragmented by incubation at 94°C for 8 min in 19.5 µl of fragmentation buffer per the manufacturer′s instructions (Illumina, San Diego, CA). Sequencing libraries were prepared using an Illumina TruSeq stranded-RNA kit, version 2, following the manufacturer’s protocol. The indexed samples were sequenced on a single lane of an Illumina HiSeq 1500 using the 2 × 50 paired-end protocol. The resulting BCL files were converted to fastq files using Illumina bcl2fastq2 software, version 2.17. Reads were checked for quality using FastQC and aligned to the *Y. pestis* CO92 genome using Burrows-Wheeler Aligner (BWA) via Illumina BaseSpace. Transcript counts were generated using the Bioconductor GenomicAlignments package (42), and differential expression was determined using DESeq2 (43). Euclidean distance mapping was performed using the distance function in R from the regularized-logarithm-transformed counts. Poisson distance mapping was performed using the method described by Witten (44), and heat maps were generated in R.

### qRT-PCR.

Quantitative real-time PCR (qRT-PCR) was performed using the QuantiFast Sybr green PCR kit (Qiagen), according to the manufacturer’s protocol, and a 7300 real-time PCR system (Applied Biosystems, Grand Island, NY). Experimental gene mRNA levels were corrected to the level of 16S rRNA, and relative levels were calculated in relation to mRNA levels in the WT CO92 control. The results shown are the averages and standard deviations from three experiments. RNA samples were quantified using a NanoDrop spectrophotometer (NanoDrop Technologies, Wilmington, DE) and qualified by analysis on an RNA nano chip using the Agilent 2100 Bioanalyzer (Agilent Technologies, Santa Clara, CA). Synthesis of cDNA was performed with 0.5 µg of total RNA in a 20-µl reaction mixture using the reagents in the TaqMan reverse transcription reagent kit from Applied Biosystems. The reaction conditions were as follow: 10 min at 25°C, 30 min at 48°C, and 5 min at 95°C. PCR amplifications were performed using 5 µl of cDNA in a total volume of 50 µl FailSafe buffer C (Epicenter Biotechnologies, Madison, WI).

### Western blotting for T3SS effectors.

Western blotting for *Y. pestis*-secreted T3SS effectors was performed as previously described (40). In brief, overnight cultures of *Y. pestis* CO92 or its mutants, grown in HIB at 28°C, were diluted 1:20 in 5 ml HIB supplemented with 5 mM EGTA to trigger the low-calcium response. The cultures were incubated at 28°C for 2 h before being shifted to 37°C (to activate the T3SS) for an additional 3 h of growth. Supernatants were precipitated with 20% (vol/vol) trichloroacetic acid (TCA) on ice for 2 h. The TCA precipitates were then washed and dissolved in SDS-PAGE buffer and analyzed by immunoblotting using antibodies to YopE or LcrV (Santa Cruz Biotechnology, Santa Cruz, CA). The secondary antibodies were anti-rabbit IgG or anti-mouse IgG as appropriate (Southern Biotech, Birmingham, AL). Blots were developed using SuperSignal West Dura (Pierce Biotechnology, Thermo Fisher Scientific, Waltham, MA). Protein-loading normalization was accomplished through visualization of total protein in blots using stain-free technology (Bio-Rad, Hercules, CA).

### Pla protease activity.

Pla protease activity measurement was performed as previously described (40). In brief, bacteria were grown as described above for AI-2 analysis and collected at the time of maximal AI-2 level. Cultures were centrifuged, washed twice, and resuspended in PBS to obtain a final OD_600_ of 0.1 using a spectrophotometer (SmartSpec 300; Bio-Rad). For each sample, 50-μl suspensions were added to wells of a black microtiter plate (Costar Corning, Inc., Corning, NY) in triplicate. The hexapeptide substrate dabcyl-Arg-Arg-Ile-Asn-Arg-Glu-(edans)-NH_2_, synthesized on Sieber amide resin (45), was added to the wells at a final concentration of 2.5 μg/50 μl. The kinetics of substrate cleavage by Pla was measured every 10 min for 3 h by a fluorometric assay (excitation/emission wavelengths, 360/460 nm) at 37°C on a BioTek Synergy HT spectrophotometer (BioTek Instruments, Inc., Winooski, VT).
