# Supplementary figures and images for: New Insights into Autoinducer-2 Signaling as a Virulence Regulator in a Mouse Model of Pneumonic Plague
Source: mSphere. 2016 Dec 14;1(6):e00342-16. doi: 10.1128/mSphere.00342-16 (PMC5156673; doi:10.1128/mSphere.00342-16)

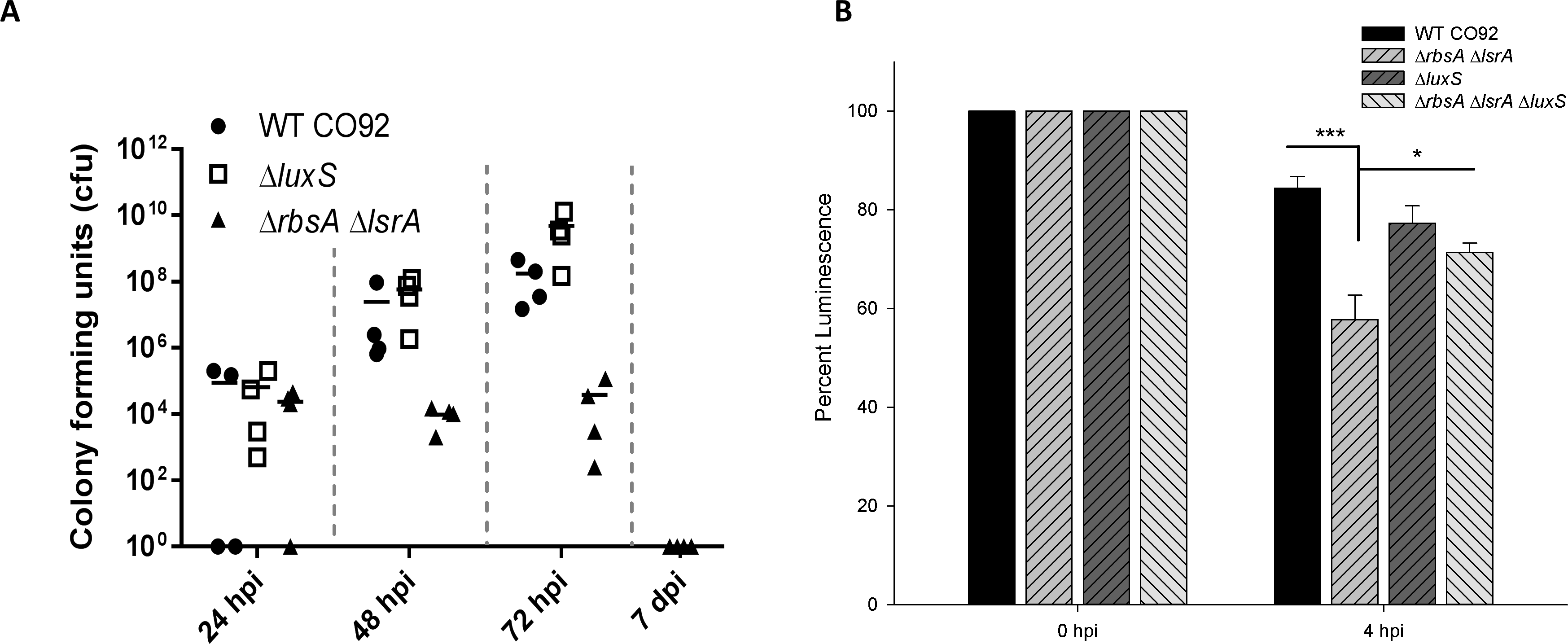

Supplement: Figure S1 [file sph006162209sf1.tif]

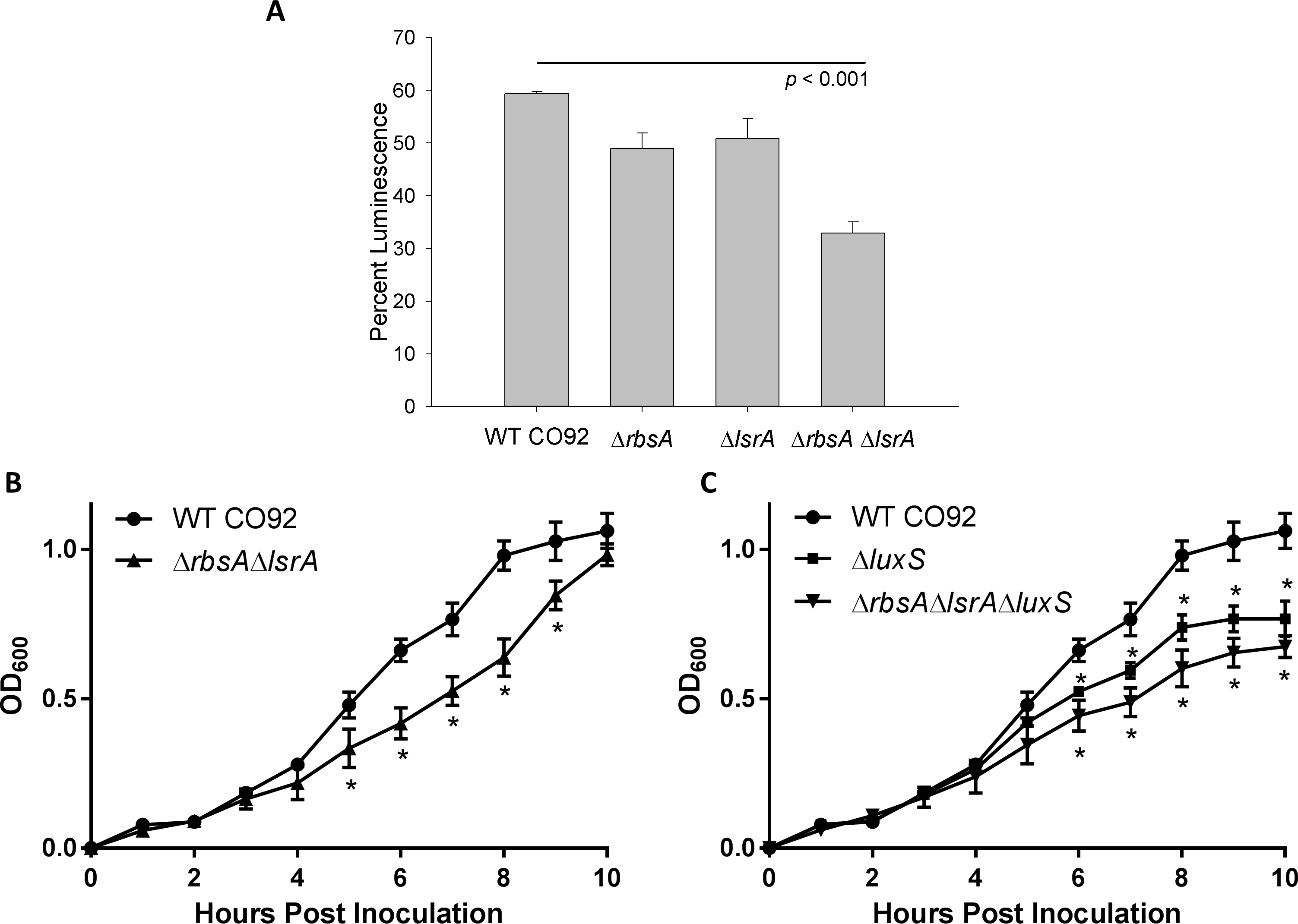

Supplement: Figure S2 [file sph006162209sf2.tif]

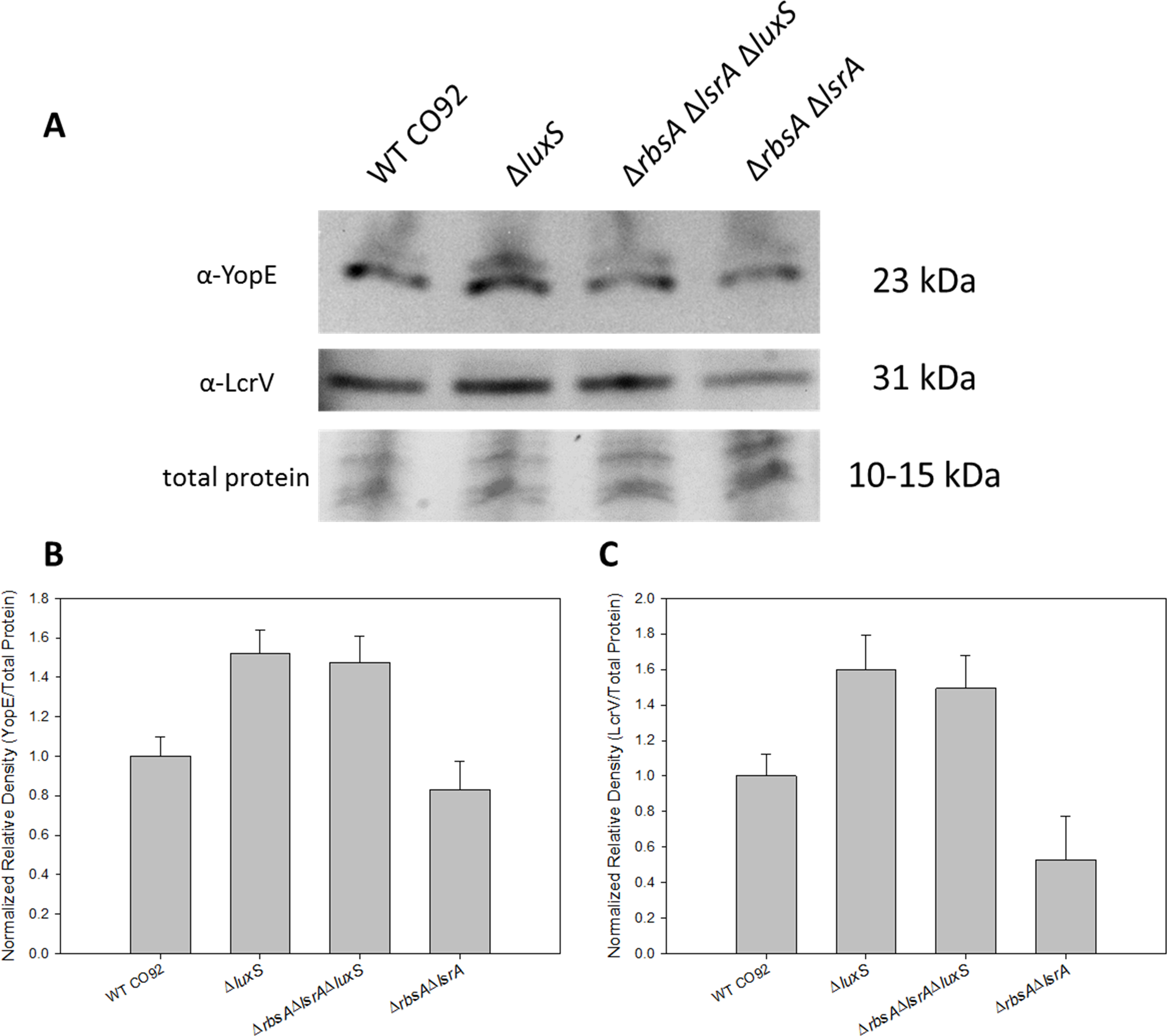

Supplement: Figure S3 [file sph006162209sf3.tif]

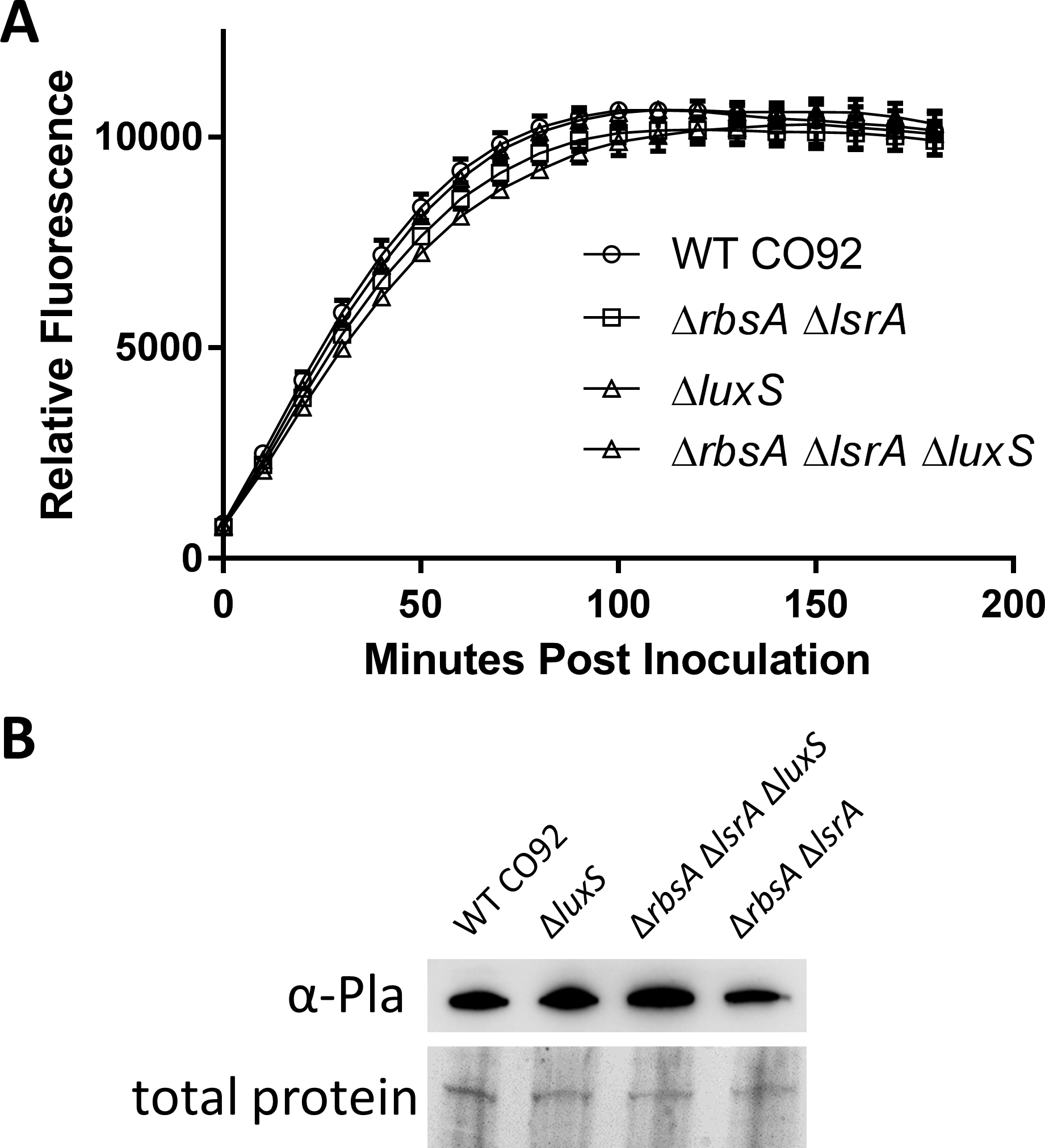

Supplement: Figure S4 [file sph006162209sf4.tif]
