# Supplementary material for: New Insights into Autoinducer-2 Signaling as a Virulence Regulator in a Mouse Model of Pneumonic Plague
Source: mSphere. 2016 Dec 14;1(6):e00342-16. doi: 10.1128/mSphere.00342-16 (PMC5156673; doi:10.1128/mSphere.00342-16)
Supplement: Table S1 [file sph006162209st5.pdf]

ΔrbsA ΔlsrA vs. WT CO92			
Gene Symbol	log fold change	p_{adj}	Genome Annotation
accC	-0.627	5.563E-02	acetyl-CoA carboxylase biotin carboxylase subunit
adhE	-0.577	2.944E-04	bifunctional acetaldehyde-CoA/alcohol dehydrogenase
ansA	-0.926	1.787E-07	cytoplasmic asparaginase I
araC	-0.573	2.417E-02	DNA-binding transcriptional regulator AraC
araF	-0.573	2.417E-02	L-arabinose-binding protein
araG	-0.573	2.417E-02	L-arabinose transporter ATP-binding protein
arnT	1.367	1.599E-23	4-amino-4-deoxy-L-arabinose transferase
atpF	-0.485	4.442E-02	ATP synthase F0F1 subunit B
atpH	0.592	3.478E-03	ATP synthase F0F1 subunit delta
bioD	0.438	5.255E-02	dithiobiotin synthetase
btuE	1.472	8.976E-18	glutathione peroxidase
ccmA	0.591	6.228E-02	cytochrome c biogenesis protein CcmA
ccmD	-0.549	9.220E-02	heme exporter protein D
ccmE	-0.549	9.220E-02	cytochrome c-type biogenesis protein CcmE
ccmF	-0.549	9.220E-02	cytochrome c-type biogenesis protein
ccmG	-0.549	9.220E-02	thiol:disulfide interchange protein DsbE
ccmH	-0.549	9.220E-02	cytochrome c-type biogenesis protein
clpB	0.663	7.864E-03	Clp ATPase
cls	-1.077	1.771E-08	cardiolipin synthetase
cpxP	1.025	8.999E-07	periplasmic stress adaptor protein CpxP
csrB	-0.434	2.173E-02	#N/A
cysT	-0.841	6.566E-04	sulfate/thiosulfate transporter subunit
dadA	-1.291	8.502E-15	D-amino acid dehydrogenase small subunit
dksA	1.317	8.477E-09	RNA polymerase-binding transcription factor
dnaJ	-0.335	5.012E-02	molecular chaperone DnaJ
dnaK	-0.335	5.012E-02	molecular chaperone DnaK
dps	2.556	9.163E-47	DNA starvation/stationary phase protection protein Dps
fis	1.189	1.003E-07	Fis family transcriptional regulator
fklB	1.189	1.003E-07	peptidyl-prolyl cis-trans isomerase
flgG	-0.772	2.039E-04	flagellar basal body rod protein FlgG
fliH	-0.767	5.874E-02	flagellar assembly protein H
ftn	1.218	7.921E-07	ferritin
gapA	-0.762	1.757E-04	glyceraldehyde 3-phosphate dehydrogenase A
glnP	1.683	2.126E-13	glutamine ABC transporter permease
glnQ	1.449	1.671E-12	glutamine ABC transporter ATP-binding protein
greA	1.343	1.849E-11	transcription elongation factor GreA

"-" indicates a down regulation at the indicated log fold change

hcaT	-0.973	1.249E-03	3-phenylpropionic acid transporter
hdeB	1.457	2.376E-20	acid-resistance protein
htpG	0.704	1.330E-03	heat shock protein 90
ibpA	0.377	8.126E-02	heat shock protein IbpA
ibpB	0.377	8.126E-02	heat shock chaperone IbpB
ihfA	0.379	9.252E-02	integration host factor subunit alpha
ihfB	1.114	2.313E-08	integration host factor subunit beta
infC	1.767	2.558E-12	translation initiation factor IF-3
irp1	-1.719	9.119E-07	yersiniabactin biosynthetic protein
irp2	-1.081	7.403E-04	yersiniabactin biosynthetic protein
irp3	-1.375	4.403E-03	yersiniabactin biosynthetic protein YbtU
lplA	1.579	4.750E-24	lipoate-protein ligase A
marC	1.715	5.326E-23	multiple drug resistance protein MarC
menF	-0.710	4.296E-02	menaquinone-specific isochorismate synthase
mipB	-1.088	4.479E-04	fructose-6-phosphate aldolase
mrpA	1.219	1.572E-06	mannose-resistant fimbrial protein
napA	1.109	4.581E-08	nitrate reductase catalytic subunit
napB	1.109	4.581E-08	citrate reductase cytochrome c-type subunit
napC	1.109	4.581E-08	cytochrome c-type protein NapC
napD	1.109	4.581E-08	assembly protein for periplasmic nitrate reductase
napF	1.109	4.581E-08	ferredoxin-type protein NapF
nhaB	-1.284	1.417E-11	sodium/proton antiporter
nirB	0.991	2.197E-05	nitrite reductase
nqrB	-0.710	2.471E-04	Na(+)-translocating NADH-quinone reductase subunit B
nqrC	-0.710	2.471E-04	Na(+)-translocating NADH-quinone reductase subunit C
nqrD	-0.710	2.471E-04	Na(+)-translocating NADH-quinone reductase subunit D
nudG	-1.433	5.901E-05	pyrimidine (deoxy)nucleoside triphosphate pyrophosphohydrolase
opdA	1.014	6.365E-09	oligopeptidase A
oppF	-1.172	1.010E-06	oligopeptide transport ATP-binding protein
pheS	0.805	3.318E-06	phenylalanyl-tRNA synthetase subunit alpha
pheT	0.520	1.202E-02	phenylalanyl-tRNA synthetase subunit beta
pmrF	1.517	8.834E-21	undecaprenyl phosphate 4-deoxy-4-formamido-L-arabinose transferase
priB	0.550	6.102E-04	primosomal replication protein N
ptsG	0.570	1.894E-03	PTS system glucose-specific transporter subunits IIBC
rbsA	-5.047	2.214E-47	sugar transport system ATP-binding protein
rhaA	-1.239	4.257E-03	L-rhamnose isomerase

"-" indicates a down regulation at the indicated log fold change

rplS	1.211	5.847E-05	50S ribosomal protein L19
rplT	1.757	5.262E-05	50S ribosomal protein L20
rpmG	0.941	2.788E-04	50S ribosomal protein L33
rpmI	1.424	3.513E-13	50S ribosomal protein L35
selD	-1.626	1.057E-18	selenophosphate synthetase
solA	1.818	5.053E-26	N-methyltryptophan oxidase
tauA	-0.487	7.032E-02	taurine transporter substrate binding subunit
tauD	-0.644	4.019E-02	taurine dioxygenase
tdk	-1.528	2.575E-17	thymidine kinase
thrS	1.075	1.749E-05	threonyl-tRNA synthetase
tig	-0.347	3.181E-02	trigger factor
tonB	-1.690	9.903E-18	transport protein TonB
topB	-1.048	3.311E-11	DNA topoisomerase III
tpiA	1.390	4.471E-08	triosephosphate isomerase
tqsA	1.683	1.264E-27	transport protein
xylF	0.829	6.203E-07	D-xylose transporter subunit XylF
yecC	-0.694	8.225E-04	amino-acid ABC transporter ATP-binding protein YecC
yecS	-0.780	2.231E-03	amino-acid ABC transporter permease
yfeA	1.965	3.266E-40	substrate-binding protein
yfeB	1.915	8.948E-35	ATP-binding transport protein
yfeC	1.357	7.281E-10	chelated iron transport system membrane protein
yfeD	1.188	4.872E-10	chelated iron transport system membrane protein
yfeE	2.343	2.143E-42	yfeABCD locus regulator
yfgD	0.471	6.103E-03	arsenate reductase
yhjA	-0.544	5.237E-02	cytochrome C peroxidase
yopQ	1.523	1.731E-13	Yop targeting protein (plasmid)
ypeI	2.192	5.649E-23	N-acylhomoserine lactone synthase
ypeR	2.140	1.284E-24	quorum-sensing transcriptional activator YpeR
YPMT1.01	0.445	6.360E-03	putative transposase (plasmid)
YPMT1.04c	-0.743	2.885E-04	putative phage tail protein (plasmid)
YPMT1.11c	-1.623	3.547E-05	hypothetical protein YPMT1.11c (plasmid)
YPMT1.35c	-1.517	3.050E-05	hypothetical protein YPMT1.35c (plasmid)
YPMT1.45c	1.201	1.329E-02	hypothetical protein YPMT1.45c (plasmid)
YPMT1.46c	0.874	7.318E-02	hypothetical protein YPMT1.46c (plasmid)
YPMT1.55c	1.759	1.871E-11	hypothetical protein YPMT1.55c (plasmid)
YPMT1.59c	-1.801	5.220E-05	putative DNA-binding protein (plasmid)
YPMT1.75c	-1.164	1.168E-02	reverse transcriptase (plasmid)
YPMT1.79c	-1.553	1.373E-11	transposase (plasmid)
YPO0127	0.386	7.035E-02	DNA uptake protein
YPO0147	-1.185	4.057E-04	hypothetical protein YPO0147
YPO0148	-1.459	5.842E-04	hypothetical protein YPO0148
YPO0285	0.970	1.914E-04	hypothetical protein YPO0285

"-" indicates a down regulation at the indicated log fold change

YPO0286	0.970	1.914E-04	coproporphyrinogen III oxidase
YPO0397	-1.125	8.544E-04	hypothetical protein YPO0397
YPO0400	0.713	6.441E-06	hypothetical protein YPO0400
YPO0407	1.857	3.011E-18	autoinducer-2 (AI-2) modifying protein LsrG
YPO0412	-2.897	1.465E-26	ABC transporter ATP-binding protein
YPO0624	1.246	1.053E-07	hypothetical protein YPO0624
YPO0625	1.468	4.344E-13	hypothetical protein YPO0625
YPO0626	1.080	8.688E-07	hypothetical protein YPO0626
YPO0627	1.193	1.744E-10	translational inhibitor protein
YPO0640	0.721	2.858E-04	hypothetical protein YPO0640
YPO0950	1.249	4.336E-07	hypothetical protein YPO0950
YPO1316	-0.628	3.200E-02	iron/ascorbate oxidoreductase family protein
YPO1318	-1.197	5.867E-04	ABC transporter ATP-binding protein
YPO1500	1.277	1.320E-04	hypothetical protein YPO1500
YPO1718	1.039	8.337E-09	hypothetical protein YPO1718
YPO1736	1.603	2.242E-14	hypothetical protein YPO1736
YPO1941	1.377	2.161E-11	hypothetical protein YPO1941
YPO1942	1.066	3.542E-10	hypothetical protein YPO1942
YPO1993	-2.104	2.585E-17	dehydrogenase
YPO1994	-1.270	5.646E-07	hypothetical protein YPO1994
YPO1995	-1.418	7.043E-10	hypothetical protein YPO1995
YPO1996	-1.084	1.938E-05	hypothetical protein YPO1996
YPO2055	1.039	1.040E-06	hypothetical protein YPO2055
YPO2095	1.535	1.616E-04	hypothetical protein YPO2095
YPO2096	1.455	9.429E-09	hypothetical protein YPO2096
YPO2128	1.112	6.652E-04	phage-like lipoprotein
YPO2137	-1.827	6.540E-13	hypothetical protein YPO2137
YPO2138	-1.550	3.909E-04	aminotransferase
YPO2139	-1.147	3.372E-02	hypothetical protein YPO2139
YPO2148	-1.779	1.543E-14	multidrug resistance protein
YPO2151	-1.265	1.799E-06	hypothetical protein YPO2151
YPO2152	-1.160	7.955E-08	hypothetical protein YPO2152
YPO2153	-1.086	1.172E-06	hypothetical protein YPO2153
YPO2163	-1.563	1.398E-08	hypothetical protein YPO2163
YPO2169	-3.199	9.937E-18	LysR family transcriptional regulator
YPO2172	-1.422	3.911E-09	hypothetical protein YPO2172
YPO2173	-1.707	7.411E-17	response regulator of RpoS
YPO2192	-1.229	4.457E-05	hypothetical protein YPO2192
YPO2398	1.270	3.174E-11	murein L,D-transpeptidase
YPO2400	1.044	7.715E-07	bifunctional cysteine desulfurase/selenocysteine lyase
YPO2410	1.366	5.855E-09	hypothetical protein YPO2410
YPO2416	1.418	1.912E-11	hypothetical protein YPO2416
YPO2419	1.561	2.012E-14	hypothetical protein YPO2419

"-" indicates a down regulation at the indicated log fold change

YPO2420	1.788	1.193E-23	bifunctional UDP-glucuronic acid decarboxylase/UDP-4-amino-4-deoxy-L-arabinose formyltransferase
YPO2422	1.456	4.609E-16	UDP-4-amino-4-deoxy-L-arabinose--oxoglutarate aminotransferase
YPO2426	1.465	8.920E-11	hypothetical protein YPO2426
YPO2434	1.924	2.877E-17	hypothetical protein YPO2434
YPO2436	1.779	5.095E-16	hypothetical protein YPO2436
YPO2446	1.355	1.352E-06	2-deoxyglucose-6-phosphatase
YPO2449	1.641	2.255E-24	LuxR family transcriptional regulator
YPO2451	1.077	1.802E-08	hypothetical protein YPO2451
YPO2455	1.004	1.698E-03	hypothetical protein YPO2455
YPO2459	1.057	6.961E-03	transporter protein
YPO2462	1.274	6.821E-05	hypothetical protein YPO2462
YPO2464	1.256	8.559E-06	hypothetical protein YPO2464
YPO2465	1.353	2.932E-12	hypothetical protein YPO2465
YPO2467	1.114	5.388E-07	hypothetical protein YPO2467
YPO2470	1.102	7.158E-03	hypothetical protein YPO2470
YPO2476	1.280	4.455E-05	sugar ABC transporter permease
YPO2481	1.840	3.343E-10	hypothetical protein YPO2481
YPO2482	1.075	1.215E-03	hypothetical protein YPO2482
YPO2483	1.705	2.175E-10	hypothetical protein YPO2483
YPO2484	1.474	1.563E-03	hypothetical protein YPO2484
YPO2489	1.821	3.770E-15	hypothetical protein YPO2489
YPO2490	1.076	1.217E-07	hemolysin
YPO2494	1.173	3.285E-04	transporter
YPO2495	1.254	2.028E-04	hypothetical protein YPO2495
YPO2496	1.004	9.254E-04	tartrate dehydrogenase
YPO2498	1.724	2.481E-23	LacI family transcriptional regulator
YPO2504	1.781	7.524E-10	hypothetical protein YPO2504
YPO2511	2.530	8.532E-30	hypothetical protein YPO2511
YPO2515	1.317	1.897E-10	chemotactic transducer
YPO2563	1.253	5.064E-10	hypothetical protein YPO2563
YPO2590	1.014	8.688E-04	hypothetical protein YPO2590
YPO2675	1.124	1.830E-06	voltage-gated potassium channel
YPO2855	1.286	1.184E-08	protease
YPO3048	1.226	4.067E-11	ABC transporter ATP-binding protein
YPO3050	1.037	3.082E-05	hypothetical protein YPO3050
YPO3121	1.093	5.580E-04	hypothetical protein YPO3121
YPO3136	1.165	3.015E-06	hypothetical protein YPO3136
YPO3170	1.286	7.196E-08	nucleotide-binding protein
YPO3213	1.043	1.584E-06	hypothetical protein YPO3213
YPO3343	1.112	1.496E-05	ABC transporter substrate-binding protein
YPO3387	-1.042	4.758E-07	iron-sulfur cluster insertion protein ErpA
YPO3518	-1.282	3.111E-06	hypothetical protein YPO3518
YPO3617	1.460	9.233E-12	hypothetical protein YPO3617
YPO3618	1.159	1.646E-05	oxidoreductase

"-" indicates a down regulation at the indicated log fold change

YPO3655	1.004	9.815E-08	tRNA-dihydrouridine synthase B
YPO3681	-2.421	4.626E-31	insecticial toxin
YPO3682	-3.170	2.008E-29	LysR family transcriptional regulator
YPO3784	1.070	1.860E-09	carbon starvation protein
YPO3874	1.126	4.400E-05	hypothetical protein YPO3874
YPO3908	1.027	2.065E-05	periplasmic protein
YPO3957	-1.099	2.424E-02	hypothetical protein YPO3957
YPO3967	1.042	1.991E-05	phosphate transport protein
YPO4050	1.129	5.580E-04	hypothetical protein YPO4050
YPO4109	-1.055	1.197E-04	amino acid transport system permease
YPO4110	-1.527	1.173E-07	ABC transporter permease
YPPCP1.02	1.364	7.389E-03	transposase/IS protein (plasmid)
YPPCP1.06	1.028	4.144E-02	hypothetical protein YPPCP1.06 (plasmid)
YPt_02	-1.087	4.383E-02	#N/A
YPt_29	-1.267	2.096E-04	#N/A
YPt_53	1.252	2.982E-03	#N/A
YPt_63	-1.246	2.279E-03	#N/A
zntA	-1.087	2.797E-05	zinc/cadmium/mercury/lead-transporting ATPase

Δ*luxS* vs. WT CO92

Gene Symbol	log fold change	p_{adj}	Genome Annotation
aceA	1.309	4.894E-09	isocitrate lyase
acpD	1.843	9.745E-23	azoreductase
ansA	-1.069	1.894E-07	cytoplasmic asparaginase I
arnT	1.107	4.376E-12	4-amino-4-deoxy-L-arabinose transferase
astA	1.067	1.017E-02	arginine succinyltransferase
cls	-1.058	2.354E-06	cardiolipin synthetase
cysP	-1.272	3.853E-08	thiosulfate transporter subunit
dps	1.236	1.019E-08	DNA starvation/stationary phase protection protein Dps
dsbB	-1.202	2.045E-07	disulfide bond formation protein B
fadA	1.069	3.096E-08	3-ketoacyl-CoA thiolase
fadB	1.037	4.423E-09	multifunctional fatty acid oxidation complex subunit alpha
fliY	-1.124	5.495E-08	cystine transporter subunit
gapA	-1.069	2.380E-06	glyceraldehyde 3-phosphate dehydrogenase A
glnP	1.614	7.424E-10	glutamine ABC transporter permease
glnQ	1.341	1.822E-08	glutamine ABC transporter ATP-binding protein
gptB	1.126	2.338E-02	PTS system mannose-specific transporter subunit IIAB
hslV	-1.104	2.416E-05	ATP-dependent protease peptidase subunit
infC	1.240	5.295E-05	translation initiation factor IF-3

"-" indicates a down regulation at the indicated log fold change

irp1	-1.034	2.541E-02	yersiniabactin biosynthetic protein
irp3	-1.261	2.292E-02	yersiniabactin biosynthetic protein YbtU
irp4	-1.374	1.168E-02	yersiniabactin biosynthetic protein YbtT
lplA	1.160	3.422E-10	lipoate-protein ligase A
luxS	-8.422	8.167E-205	S-ribosylhomocysteinase
mtlK	1.012	2.554E-03	mannitol transport ATP-binding protein
nhaB	-1.148	2.344E-07	sodium/proton antiporter
nudG	-1.198	6.031E-03	pyrimidine (deoxy)nucleoside triphosphate pyrophosphohydrolase
ompF	-1.125	8.481E-08	porin
pmrF	1.442	8.276E-15	undecaprenyl phosphate 4-deoxy-4-formamido-L-arabinose transferase
pncA	-1.000	6.487E-04	nicotinamidase/pyrazinamidase
ppsA	1.335	1.197E-15	phosphoenolpyruvate synthase
pspG	-1.190	1.544E-02	phage shock protein G
purK	-1.151	2.231E-02	phosphoribosylaminoimidazole carboxylase ATPase subunit
rhaA	-1.092	4.774E-02	L-rhamnose isomerase
rimI	-1.055	1.179E-02	ribosomal-protein-alanine N-acetyltransferase
selD	-1.140	1.891E-07	selenophosphate synthetase
solA	1.248	1.218E-09	N-methyltryptophan oxidase
sufA	1.069	5.099E-07	iron-sulfur cluster assembly scaffold protein
sufC	1.349	3.333E-10	cysteine desulfurase
tam	1.071	2.483E-04	trans-aconitate 2-methyltransferase
tauA	-1.177	1.342E-03	taurine transporter substrate binding subunit
tdk	-1.048	1.268E-06	thymidine kinase
tonB	-1.156	8.408E-07	transport protein TonB
tpiA	1.020	1.408E-03	triosephosphate isomerase
xthA	-1.107	1.036E-05	exonuclease III
yecS	-1.320	2.568E-06	amino-acid ABC transporter permease
yfeA	1.287	1.948E-13	substrate-binding protein
yfeB	1.279	3.618E-12	ATP-binding transport protein
yfeE	1.331	7.091E-11	yfeABCD locus regulator
yopR	1.109	1.157E-05	secreted protein (plasmid)
ypeI	1.181	1.743E-05	N-acylhomoserine lactone synthase
ypeR	1.262	5.750E-07	quorum-sensing transcriptional activator YpeR
YPO0148	-1.116	3.959E-02	hypothetical protein YPO0148
YPO0407	1.102	2.813E-05	autoinducer-2 (AI-2) modifying protein LsrG
YPO0419	1.043	3.409E-03	hypothetical protein YPO0419
YPO0435	-1.023	7.075E-03	Na+ dependent nucleoside transporter family protein
YPO0623	1.370	1.197E-15	aminotransferase

"-" indicates a down regulation at the indicated log fold change

YPO0624	1.788	3.618E-12	hypothetical protein YPO0624
YPO0625	1.813	1.171E-15	hypothetical protein YPO0625
YPO0626	1.305	1.569E-07	hypothetical protein YPO0626
YPO0627	1.226	1.401E-08	translational inhibitor protein
YPO1061	1.045	2.231E-02	hypothetical protein YPO1061
YPO1096	1.141	8.865E-05	hypothetical protein YPO1096
YPO1409	1.122	1.893E-07	metallo-beta-lactamase superfamily protein
YPO1465	-1.179	4.446E-02	hypothetical protein YPO1465
YPO1941	1.084	6.858E-07	hypothetical protein YPO1941
YPO1975	1.371	4.188E-04	hypothetical protein YPO1975
YPO2137	-1.265	2.581E-06	hypothetical protein YPO2137
YPO2138	-1.639	3.091E-04	aminotransferase
YPO2139	-1.609	2.075E-03	hypothetical protein YPO2139
YPO2140	-1.149	3.579E-07	hypothetical protein YPO2140
YPO2158	-1.378	6.733E-06	methionine sulfoxide reductase B
YPO2163	-1.333	3.779E-06	hypothetical protein YPO2163
YPO2169	-2.648	1.132E-12	LysR family transcriptional regulator
YPO2171	-1.069	5.444E-08	formyltetrahydrofolate deformylase
YPO2172	-1.341	8.865E-08	hypothetical protein YPO2172
YPO2313	1.235	3.667E-03	hypothetical protein YPO2313
YPO2398	1.176	3.448E-09	murein L,D-transpeptidase
YPO2400	1.127	1.569E-07	bifunctional cysteine desulfurase/selenocysteine lyase
YPO2401	1.302	2.803E-10	cysteine desulfurase
YPO2403	1.095	9.086E-07	cysteine desulfurase
YPO2406	1.002	9.330E-05	hypothetical protein YPO2406
YPO2419	1.400	3.947E-11	hypothetical protein YPO2419
YPO2420	1.548	3.544E-17	bifunctional UDP-glucuronic acid decarboxylase/UDP-4-amino-4-deoxy-L-arabinose formyltransferase
YPO2422	1.223	6.020E-11	UDP-4-amino-4-deoxy-L-arabinose--oxoglutarate aminotransferase
YPO2426	1.233	2.244E-07	hypothetical protein YPO2426
YPO2438	1.211	2.206E-06	membrane-bound lytic murein transglycosylase
YPO2446	1.011	1.371E-03	2-deoxyglucose-6-phosphatase
YPO2449	1.313	2.824E-15	LuxR family transcriptional regulator
YPO2462	1.278	1.236E-04	hypothetical protein YPO2462
YPO2464	1.060	5.847E-04	hypothetical protein YPO2464
YPO2465	1.106	7.137E-08	hypothetical protein YPO2465
YPO2476	1.033	3.446E-03	sugar ABC transporter permease
YPO2478	1.048	6.927E-05	LacI family transcriptional regulator
YPO2481	1.542	6.482E-07	hypothetical protein YPO2481
YPO2482	1.025	4.252E-03	hypothetical protein YPO2482
YPO2483	1.493	1.385E-07	hypothetical protein YPO2483
YPO2484	1.450	3.516E-03	hypothetical protein YPO2484

"-" indicates a down regulation at the indicated log fold change

YPO2489	1.601	2.742E-11	hypothetical protein YPO2489
YPO2498	1.270	3.071E-12	LacI family transcriptional regulator
YPO2504	1.219	2.133E-04	hypothetical protein YPO2504
YPO2511	1.900	2.081E-16	hypothetical protein YPO2511
YPO2590	1.044	1.083E-03	hypothetical protein YPO2590
YPO3681	-2.388	2.239E-30	insecticial toxin
YPO3682	-3.732	5.334E-38	LysR family transcriptional regulator
YPO4109	-1.074	1.712E-04	amino acid transport system permease
YPO4110	-1.624	4.817E-08	ABC transporter permease
YPO4111	-1.346	7.349E-13	substrate-binding protein
YPt_02	-1.331	1.544E-02	#N/A
YPt_03	-1.186	8.469E-03	#N/A
YPt_16	-1.012	6.261E-03	#N/A
YPt_26	-1.131	8.567E-03	#N/A
YPt_29	-1.769	1.931E-07	#N/A
YPt_34	-1.286	8.443E-03	#N/A
YPt_40	-1.682	1.650E-07	#N/A
YPt_43	-1.175	2.266E-03	#N/A
YPt_44	-1.365	6.380E-03	#N/A
YPt_55	-1.122	4.061E-02	#N/A
YPt_59	-1.415	2.037E-04	#N/A
YPt_63	-1.183	7.458E-03	#N/A

"-" indicates a down regulation at the indicated log fold change
