# Supplementary material for: New Insights into Autoinducer-2 Signaling as a Virulence Regulator in a Mouse Model of Pneumonic Plague
Source: mSphere. 2016 Dec 14;1(6):e00342-16. doi: 10.1128/mSphere.00342-16 (PMC5156673; doi:10.1128/mSphere.00342-16)
Supplement: Table S2 [file sph006162209st6.pdf]

ΔluxS vs. *ΔrbsA ΔlsrA ΔluxS*			
**Gene Symbol**	**log fold change**	***p* adj**	**Genome Annotation**
caf1A	-1.978	1.129E-27	putative F1 capsule anchoring protein (plasmid)
clpB	1.212	1.489E-05	Clp ATPase
ddg	-1.056	3.674E-03	lipid A biosynthesis palmitoleoyl acyltransferase
dnaJ	1.037	9.843E-07	molecular chaperone DnaJ
dnaK	1.208	5.107E-08	molecular chaperone DnaK
dps	1.320	2.453E-09	DNA starvation/stationary phase protection protein Dps
hslU	1.256	3.388E-07	ATP-dependent protease ATP-binding subunit HslU
htpG	1.155	3.626E-08	heat shock protein 90
ibpA	1.365	9.097E-10	heat shock protein IbpA
lpp	1.092	8.362E-04	major outer membrane lipoprotein
mrpA	1.240	1.149E-04	mannose-resistant fimbrial protein
psaA	1.167	8.906E-05	pH 6 antigen (antigen 4) (adhesin)
psaE	1.229	5.954E-07	regulatory protein
psaF	1.681	1.075E-08	hypothetical protein YPO1302
rbsA	-5.340	9.187E-50	sugar transport system ATP-binding protein
rpsO	1.004	2.397E-03	30S ribosomal protein S15
sbp1	1.007	4.501E-07	sulfate transporter subunit
sopB	1.039	2.041E-06	plasmid-partitioning protein (plasmid)
yfeE	1.012	4.158E-06	yfeABCD locus regulator
yfiA	1.273	3.626E-08	sigma 54 modulation protein
yopQ	1.251	6.978E-07	Yop targeting protein (plasmid)
ypeI	1.011	7.592E-04	N-acylhomoserine lactone synthase
YPMT1.06c	-1.032	4.392E-04	host specificity protein J (plasmid)
YPMT1.32	-1.109	1.389E-02	putative lipoprotein (plasmid)
YPMT1.33	-1.046	3.563E-02	putative transcriptional regulator (plasmid)
YPMT1.34A	1.312	4.626E-03	hypothetical protein YPMT1.34A (plasmid)
YPMT1.54	1.042	4.984E-05	hypothetical protein YPMT1.54 (plasmid)
YPMT1.55c	1.656	3.316E-07	hypothetical protein YPMT1.55c (plasmid)
YPMT1.66c	-1.108	1.557E-05	putative DNA-binding protein (plasmid)
YPMT1.74	-1.028	2.407E-05	toxin protein (plasmid)
YPMT1.79c	-1.143	1.063E-04	transposase (plasmid)
YPMT1.84	-1.241	3.626E-08	F1 capsule antigen (plasmid)
YPO0412	-2.976	1.326E-23	ABC transporter ATP-binding protein
YPO0415	-1.055	2.288E-03	autoinducer-2 (AI-2) kinase
YPO0623	-1.080	3.810E-09	aminotransferase
YPO0882	1.308	6.455E-06	hypothetical protein YPO0882

"-" indicates a down regulation at the indicated log fold change

YPO1107	1.071	1.109E-05	heat shock protein GrpE
YPO1453	1.394	1.118E-03	hypothetical protein YPO1453
YPO2280	-1.088	5.954E-03	phage-like secreted protein
YPO2481	1.082	1.622E-03	hypothetical protein YPO2481
YPO2483	1.028	8.956E-04	hypothetical protein YPO2483
YPO2973	-1.030	3.159E-02	hypothetical protein YPO2973
YPO3527	1.179	6.828E-08	hypothetical protein YPO3527
YPO4111	1.164	4.700E-09	substrate-binding protein
YPt_06	1.487	1.216E-04	#N/A
yscA	1.306	5.452E-08	hypothetical protein YPCD1.50 (plasmid)

Δ*luxS* vs. Δ*rbsA* Δ*lsrA*

Gene Symbol	log fold change	p_{adj}	Genome Annotation
acpD	-1.342	4.649E-10	azoreductase
araC	-1.154	1.641E-04	DNA-binding transcriptional regulator AraC
araF	-2.661	8.900E-21	L-arabinose-binding protein
araG	-1.155	1.639E-04	L-arabinose transporter ATP-binding protein
atpB	1.106	9.760E-06	ATP synthase F0F1 subunit A
atpE	1.153	3.316E-05	ATP synthase F0F1 subunit C
bioD	1.481	5.759E-10	dithiobiotin synthetase
caf1A	-1.017	3.546E-06	putative F1 capsule anchoring protein (plasmid)
ccmA	1.065	1.370E-03	cytochrome c biogenesis protein CcmA
ccmF	1.380	7.286E-04	cytochrome c-type biogenesis protein
ccmG	1.573	3.590E-06	thiol:disulfide interchange protein DsbE
cpxP	1.265	1.390E-06	periplasmic stress adaptor protein CpxP
csrB	1.620	1.478E-15	#N/A
dksA	1.691	6.966E-20	RNA polymerase-binding transcription factor
efp	1.212	1.965E-11	elongation factor P
fis	1.301	9.368E-10	Fis family transcriptional regulator
gpt	1.137	9.956E-07	xanthine-guanine phosphoribosyltransferase
htpG	-1.016	1.550E-05	heat shock protein 90
ibpB	-1.022	1.397E-03	heat shock chaperone IbpB
katY	-1.379	8.379E-10	catalase-peroxidase
luxS	8.238	2.232E-185	S-ribosylhomocysteinase
menF	1.517	7.083E-05	menaquinone-specific isochorismate synthase
metK	1.049	3.830E-05	S-adenosylmethionine synthetase
mrpA	1.158	7.152E-04	mannose-resistant fimbrial protein
napA	1.599	1.888E-08	nitrate reductase catalytic subunit
napB	2.514	1.780E-12	citrate reductase cytochrome c-type subunit

"-" indicates a down regulation at the indicated log fold change

napC	1.877	1.271E-14	cytochrome c-type protein NapC
nirB	1.265	6.975E-05	nitrite reductase
poxB	-1.272	4.177E-06	pyruvate dehydrogenase
ptsG	-1.104	4.625E-07	PTS system glucose-specific transporter subunits IIBC
putP	1.017	2.451E-04	proline permease
qacE	1.518	4.466E-08	quaternary ammonium compound-resistance protein
rbsA	-4.958	3.429E-41	sugar transport system ATP-binding protein
rdgC	1.089	1.244E-07	recombination associated protein
rop	1.207	3.537E-02	putative replication regulatory protein (plasmid)
rplU	1.150	2.717E-08	50S ribosomal protein L21
rpmA	1.138	1.173E-06	50S ribosomal protein L27
rpmB	1.397	1.234E-08	50S ribosomal protein L28
rpmF	1.037	2.051E-04	50S ribosomal protein L32
rpmG	1.140	4.863E-04	50S ribosomal protein L33
rpsF	1.004	3.774E-05	30S ribosomal protein S6
rpsI	1.195	2.087E-04	30S ribosomal protein S9
tig	1.257	5.790E-13	trigger factor
ureC	-1.033	2.253E-03	urease subunit alpha
yaaH	1.353	3.316E-05	hypothetical protein YPO0467
yhjA	1.338	2.611E-06	cytochrome C peroxidase
YPCD1.01	1.193	1.349E-02	putative transposase (plasmid)
YPMT1.01	1.216	9.160E-03	putative transposase (plasmid)
YPMT1.55c	1.370	1.200E-04	hypothetical protein YPMT1.55c (plasmid)
YPMT1.58c	1.082	3.129E-02	transposase (plasmid)
YPO0285	1.454	5.931E-08	hypothetical protein YPO0285
YPO0412	-2.880	2.381E-19	ABC transporter ATP-binding protein
YPO1233	1.028	1.935E-04	prophage repressor protein
YPO1385	1.018	7.426E-04	hypothetical protein YPO1385
YPO1594	1.053	3.349E-08	hypothetical protein YPO1594
YPO1655a	-1.016	5.251E-03	#N/A
YPO1942	1.098	1.850E-08	hypothetical protein YPO1942
YPO1993	-2.788	8.551E-26	dehydrogenase
YPO1994	-2.083	4.194E-15	hypothetical protein YPO1994
YPO1995	-2.186	2.581E-19	hypothetical protein YPO1995
YPO1996	-1.831	3.128E-12	hypothetical protein YPO1996
YPO2096	1.036	8.897E-04	hypothetical protein YPO2096
YPO2148	-1.091	1.507E-04	multidrug resistance protein
YPO2173	-1.044	3.316E-05	response regulator of RpoS
YPO2282	-1.108	1.225E-04	hypothetical protein YPO2282
YPO2563	1.052	1.221E-05	hypothetical protein YPO2563
YPO2855	1.042	1.284E-04	protease
YPO3010	1.002	1.630E-03	hypothetical protein YPO3010

"-" indicates a down regulation at the indicated log fold change

YPO3170	1.390	3.472E-07	nucleotide-binding protein
YPO3617	1.082	3.210E-05	hypothetical protein YPO3617
YPO3655	1.145	7.856E-08	tRNA-dihydrouridine synthase B
YPO3784	1.075	1.293E-07	carbon starvation protein
YPO3839	-1.009	5.934E-05	hypothetical protein YPO3839
YPO3967	1.182	2.405E-05	phosphate transport protein
YPO4111	1.208	1.015E-08	substrate-binding protein
YPPCP1.01	1.041	3.687E-02	putative transposase (plasmid)
YPPCP1.02	1.520	4.676E-03	transposase/IS protein (plasmid)
YPPCP1.06	1.117	4.999E-02	hypothetical protein YPPCP1.06 (plasmid)

"-" indicates a down regulation at the indicated log fold change
