# Supplementary material for: New Insights into Autoinducer-2 Signaling as a Virulence Regulator in a Mouse Model of Pneumonic Plague
Source: mSphere. 2016 Dec 14;1(6):e00342-16. doi: 10.1128/mSphere.00342-16 (PMC5156673; doi:10.1128/mSphere.00342-16)
Supplement: Table S3 [file sph006162209st7.pdf]

Gene Symbol	log fold change	p_{adj}	Genome Annotation
ampG	-0.539	3.689E-02	muropeptide transporter
apbE	0.428	8.778E-02	thiamine biosynthesis lipoprotein
araC	-1.357	1.658E-07	DNA-binding transcriptional regulator AraC
araF	-2.523	1.644E-21	L-arabinose-binding protein
araG	-1.190	7.187E-06	L-arabinose transporter ATP-binding protein
araH	-0.980	7.083E-03	L-arabinose transporter permease
artI	0.550	9.001E-02	arginine-binding periplasmic protein 1
atpA	0.924	1.689E-07	ATP synthase F0F1 subunit alpha
atpB	0.989	7.309E-06	ATP synthase F0F1 subunit A
atpE	0.747	4.081E-03	ATP synthase F0F1 subunit C
atpF	1.131	1.638E-11	ATP synthase F0F1 subunit B
atpG	0.716	2.493E-03	ATP synthase F0F1 subunit gamma
atpH	1.160	2.716E-08	ATP synthase F0F1 subunit delta
bglA	-0.819	5.101E-05	6-phospho-beta-glucosidase
bioD	2.046	4.452E-23	dithiobiotin synthetase
bioH	-0.856	3.202E-02	biotin biosynthesis protein
bipA	0.703	3.650E-02	GTPase
carA	0.483	8.928E-02	carbamoyl phosphate synthase small subunit
carB	0.501	5.044E-02	carbamoyl phosphate synthase large subunit
ccmA	1.603	2.023E-09	cytochrome c biogenesis protein CcmA
ccmF	1.269	4.300E-04	cytochrome c-type biogenesis protein
ccmG	1.806	6.950E-10	thiol:disulfide interchange protein DsbE
cdd	-0.826	5.998E-04	cytidine deaminase
cfa	-0.588	2.184E-02	cyclopropane fatty acyl phospholipid synthase
clpX	0.379	8.276E-02	ATP-dependent protease ATP-binding subunit ClpX
coaA	0.783	6.523E-04	pantothenate kinase
cpxR	0.843	2.181E-04	DNA-binding transcriptional regulator CpxR
cru	-0.501	9.176E-02	nucleoside permease
csrB	1.514	1.500E-16	#N/A
cybB	-0.766	2.240E-04	cytochrome b561
cyoA	0.625	5.750E-03	cytochrome o ubiquinol oxidase subunit II
cyoB	0.493	2.182E-02	cytochrome O ubiquinol oxidase subunit I
cysA	-0.746	4.379E-03	sulfate/thiosulfate transporter subunit
cysB	-0.536	3.799E-02	transcriptional regulator CysB
cysI	-0.439	8.140E-02	sulfite reductase subunit beta
dcrA	0.574	4.670E-02	#N/A
dksA	1.428	2.045E-17	RNA polymerase-binding transcription factor
dxs	0.571	1.316E-02	1-deoxy-D-xylulose-5-phosphate synthase
efp	0.774	4.888E-06	elongation factor P
fabF	0.585	3.515E-02	3-oxoacyl-ACP synthase
fabG	0.512	7.677E-02	3-ketoacyl-ACP reductase
fdhD	-0.645	7.211E-02	formate dehydrogenase accessory protein
fis	1.516	1.281E-16	Fis family transcriptional regulator
fklB	1.039	8.771E-05	peptidyl-prolyl cis-trans isomerase

"-" indicates a down regulation at the indicated log fold change

fkpA	0.877	4.461E-06	FKBP-type peptidylprolyl isomerase
flgG	-1.123	4.284E-03	flagellar basal body rod protein FlgG
flhB	-0.468	6.673E-02	flagellar biosynthesis protein FlhB
focA	0.680	6.960E-03	formate transporter
gpt	0.626	4.855E-03	xanthine-guanine phosphoribosyltransferase
gyrB	0.403	4.582E-02	DNA gyrase subunit B
hflK	0.513	2.053E-02	FtsH protease regulator HflK
hpaI	-0.855	2.538E-02	2,4-dihydroxyhept-2-ene-1,7-dioic acid aldolase
ibpB	-1.749	5.391E-12	heat shock chaperone IbpB
ihfA	1.044	2.939E-06	integration host factor subunit alpha
katY	-0.958	4.813E-06	catalase-peroxidase
lemA	0.823	1.017E-02	hypothetical protein YPO2732
livJ	-0.527	8.509E-02	branched-chain amino acid-binding protein
lpxA	0.490	3.133E-02	UDP-N-acetylglucosamine acyltransferase
menF	1.119	1.138E-03	menaquinone-specific isochorismate synthase
metK	0.973	8.128E-06	S-adenosylmethionine synthetase
metN	0.649	3.133E-02	DL-methionine transporter ATP-binding protein
mglA	-0.593	1.031E-03	sugar transport ATP-binding protein
mglB	-0.737	1.640E-03	galactose-binding protein
mglC	-0.474	6.673E-02	beta-methylgalactoside transporter inner membrane protein
mltD	0.840	8.830E-06	membrane-bound lytic murein transglycosylase D
mnmC	-0.599	3.875E-02	5-methylaminomethyl-2-thiouridine methyltransferase
mntH	-0.554	2.936E-02	manganese transport protein MntH
moaA	-0.617	8.968E-02	molybdenum cofactor biosynthesis protein A
moaE	-0.780	2.839E-02	molybdopterin guanine dinucleotide biosynthesis protein MoaE
modF	-0.595	6.048E-02	molybdenum transport ATP-binding protein ModF
mrcA	0.477	3.980E-02	peptidoglycan synthetase
mtr	-0.579	6.169E-02	tryptophan-specific transport protein
mutM	0.557	6.117E-02	formamidopyrimidine-DNA glycosylase
nanT	-0.617	5.920E-02	sialic acid transporter
napA	2.030	1.281E-16	nitrate reductase catalytic subunit
napB	2.396	3.239E-14	citrate reductase cytochrome c-type subunit
napC	1.776	1.281E-16	cytochrome c-type protein NapC
ndk	0.959	2.077E-03	nucleoside diphosphate kinase
nirB	1.503	1.647E-08	nitrite reductase
nqrC	1.212	6.696E-10	Na(+)-translocating NADH-quinone reductase subunit C
nqrD	1.037	4.787E-06	Na(+)-translocating NADH-quinone reductase subunit D
nrdD	0.880	6.075E-05	anaerobic ribonucleoside triphosphate reductase

"-" indicates a down regulation at the indicated log fold change

nrdE	-0.764	1.985E-03	ribonucleotide-diphosphate reductase subunit alpha
nrdI	-0.911	9.001E-02	ribonucleotide reductase stimulatory protein
ompC	-0.975	4.813E-06	porin
ompH	0.555	2.353E-02	periplasmic chaperone
parA	-0.720	2.873E-03	partitioning protein A (plasmid)
pcnB	0.492	5.203E-02	poly(A) polymerase
pepT	0.655	5.613E-03	peptidase T
pheT	1.105	1.916E-07	phenylalanyl-tRNA synthetase subunit beta
poxB	-0.896	5.498E-04	pyruvate dehydrogenase
ppa	0.652	1.126E-04	inorganic pyrophosphatase
ppiC	0.606	2.762E-02	peptidyl-prolyl cis-trans isomerase C
prfA	0.541	3.906E-02	peptide chain release factor 1
priB	1.057	2.384E-08	primosomal replication protein N
proS	0.862	2.000E-04	prolyl-tRNA synthetase
prsA	0.811	1.878E-04	ribose-phosphate pyrophosphokinase
pstB	-0.351	4.718E-01	phosphate transporter ATP-binding protein
ptsG	-1.100	9.821E-09	PTS system glucose-specific transporter subunits IIBC
ptsH	0.856	6.141E-04	PTS system phosphohistidinoprotein-hexose phosphotransferase subunit Hpr
purF	0.502	1.112E-02	amidophosphoribosyltransferase
putP	0.730	4.463E-03	proline permease
qacE	0.955	2.322E-04	quaternary ammonium compound-resistance protein
rdgC	0.748	7.746E-05	recombination associated protein
recC	-0.384	6.254E-02	exonuclease V subunit gamma
rimM	0.491	3.689E-02	16S rRNA-processing protein RimM
rnfD	-0.528	4.362E-02	electron transport complex protein RnfD
rnhB	0.748	2.111E-03	ribonuclease HII
rodA	0.556	3.689E-02	cell wall shape-determining protein
rph	0.419	6.014E-02	ribonuclease PH
rpiA	0.581	2.330E-02	ribose-5-phosphate isomerase A
rplI	0.803	1.570E-04	50S ribosomal protein L9
rplJ	0.651	1.784E-03	50S ribosomal protein L10
rplK	0.388	9.747E-02	50S ribosomal protein L11
rplM	0.733	2.420E-04	50S ribosomal protein L13
rplU	0.568	6.227E-03	50S ribosomal protein L21
rpmA	0.921	1.342E-05	50S ribosomal protein L27
rpmB	0.953	3.077E-05	50S ribosomal protein L28
rpmF	0.675	1.056E-02	50S ribosomal protein L32
rpmH	0.662	9.150E-02	50S ribosomal protein L34
rpsF	0.760	5.384E-04	30S ribosomal protein S6
rpsJ	0.723	4.375E-03	30S ribosomal protein S10
rpsP	0.502	7.999E-03	30S ribosomal protein S16
secF	0.614	1.460E-03	preprotein translocase subunit SecF
slyD	0.468	4.545E-02	FKBP-type peptidylprolyl isomerase

"-" indicates a down regulation at the indicated log fold change

smpB	0.550	6.297E-03	SsrA-binding protein
speD	0.731	1.934E-04	S-adenosylmethionine decarboxylase
tap	-0.738	8.814E-02	RepA leader peptide Tap (plasmid)
terX	0.828	2.936E-02	tellurium resistance protein
thiI	0.572	1.763E-02	thiamine biosynthesis protein ThiI
tig	1.090	4.133E-12	trigger factor
tsf	0.538	5.613E-03	elongation factor Ts
tuf	0.572	2.982E-03	elongation factor Tu
ugpB	-0.824	4.206E-02	glycerol-3-phosphate transporter substrate-binding protein
ugpQ	-0.776	2.838E-02	cytoplasmic glycerophosphodiester phosphodiesterase
upp	0.841	2.923E-04	uracil phosphoribosyltransferase
uup	0.579	3.226E-03	ABC transporter ATPase
valS	0.511	3.004E-02	valyl-tRNA synthetase
virG	-0.483	6.644E-02	needle complex outer membrane lipoprotein precursor (plasmid)
yaaH	0.861	4.667E-03	hypothetical protein YPO0467
ybiT	0.526	7.465E-02	ABC transporter ATP-binding protein
yfgA	0.500	4.540E-02	cytoskeletal protein RodZ
yfgD	1.426	1.619E-17	arsenate reductase
yfgL	0.441	3.875E-02	outer membrane protein assembly complex subunit YfgL
yhjA	1.656	4.677E-12	cytochrome C peroxidase
yhjW	0.510	5.750E-03	phosphoethanolamine transferase
yidC	0.664	5.840E-04	inner membrane protein translocase component YidC
YPCD1.01	0.972	2.730E-02	putative transposase (plasmid)
YPCD1.92	-0.736	1.592E-02	hypothetical protein YPCD1.92 (plasmid)
YPMT1.01	1.081	8.760E-03	putative transposase (plasmid)
YPMT1.57c	0.813	4.416E-02	transposase/IS protein (plasmid)
YPMT1.58c	0.801	8.220E-02	transposase (plasmid)
YPMT1.70	-0.725	2.856E-02	putative resolvase (plasmid)
YPO0043	0.448	6.061E-02	hypothetical protein YPO0043
YPO0096	0.735	7.254E-02	transposase/IS protein
YPO0100	0.544	9.533E-02	hypothetical protein YPO0100
YPO0141	-0.593	7.755E-02	hypothetical protein YPO0141
YPO0163	-0.958	7.243E-05	hypothetical protein YPO0163
YPO0285	1.824	2.094E-15	hypothetical protein YPO0285
YPO0302	-0.523	2.313E-02	outer membrane fimbrial usher protein
YPO0327	-0.938	4.072E-02	alcohol dehydrogenase
YPO0400	1.180	1.659E-12	hypothetical protein YPO0400
YPO0507	-0.728	3.123E-02	hypothetical protein YPO0507
YPO0527	0.730	7.547E-02	transposase/IS protein
YPO0749	0.718	6.290E-03	hypothetical protein YPO0749
YPO0875	-0.998	6.606E-02	hypothetical protein YPO0875
YPO0878	-1.033	6.013E-02	regulatory protein

"-" indicates a down regulation at the indicated log fold change

YPO0900	-0.953	2.984E-03	hemolysin III
YPO0913	-0.548	8.923E-02	5-formyltetrahydrofolate cyclo-ligase
YPO0923	0.799	3.739E-02	transposase/IS protein
YPO0940	-0.482	9.445E-02	hypothetical protein YPO0940
YPO1007	-0.512	4.129E-02	hypothetical protein YPO1007
YPO1074	0.480	3.720E-02	D,D-heptose 1,7-bisphosphate phosphatase
YPO1085	0.718	8.384E-02	transposase/IS protein
YPO1237	-0.531	9.955E-02	transcriptional regulator
YPO1291	-0.779	6.668E-02	carbohydrate kinase
YPO1317	-0.536	9.316E-02	hypothetical protein YPO1317
YPO1385	0.739	6.331E-03	hypothetical protein YPO1385
YPO1426	0.680	9.150E-02	transposase/IS protein
YPO1454	-0.844	3.803E-02	(3R)-hydroxymyristoyl-ACP dehydratase
YPO1567	-0.558	3.792E-02	racemase
YPO1594	0.854	7.198E-07	hypothetical protein YPO1594
YPO1622	0.747	5.129E-02	transposase/IS protein
YPO1637	0.766	1.032E-02	hypothetical protein YPO1637
YPO1648	-0.669	5.432E-03	phosphoanhydride phosphorylase
YPO1655a	-0.618	8.305E-02	#N/A
YPO1683	0.805	2.371E-05	N-acetylmuramoyl-L-alanine amidase
YPO1684	-0.725	6.420E-02	surface protein
YPO1688	0.460	4.775E-02	hypothetical protein YPO1688
YPO1738	0.782	6.701E-03	hypothetical protein YPO1738
YPO1745a	0.792	5.980E-07	#N/A
YPO1751a	-0.494	6.535E-02	PAS/PAC domain-containing protein
YPO1942	1.066	3.542E-10	hypothetical protein YPO1942
YPO1943	0.704	2.877E-02	hypothetical protein YPO1943
YPO1946	0.735	9.342E-03	ABC transporter ATP-binding protein
YPO2025	0.787	2.991E-02	transposase/IS protein
YPO2173	-1.707	7.411E-17	response regulator of RpoS
YPO2177	0.752	5.395E-02	transposase/IS protein
YPO2228	-0.819	2.402E-04	translation initiation factor Sui1
YPO2252	-0.555	8.997E-02	toxin transport protein
YPO2262	0.463	2.831E-02	hypothetical protein YPO2262
YPO2282	-0.779	4.116E-03	hypothetical protein YPO2282
YPO2289	-0.829	6.741E-02	virulence factor
YPO2305	0.499	9.377E-03	hypothetical protein YPO2305
YPO2312	-0.437	7.736E-02	insecticidal toxin complex
YPO2517	0.793	4.234E-02	transposase/IS protein
YPO2559	0.636	4.765E-03	hypothetical protein YPO2559
YPO2560	0.604	6.502E-02	hypothetical protein YPO2560
YPO2563	1.253	5.064E-10	hypothetical protein YPO2563
YPO2568	-0.769	2.132E-02	LacI family transcriptional regulator
YPO2581	0.792	9.308E-02	sugar-binding protein
YPO2642	0.776	6.229E-02	transposase/IS protein
YPO2794	0.691	8.063E-02	hypothetical protein YPO2794
YPO2809	0.738	6.151E-02	transposase/IS protein

"-" indicates a down regulation at the indicated log fold change

YPO2811	-0.873	4.822E-02	hypothetical protein YPO2811
YPO2855	1.286	1.184E-08	protease
YPO2873	0.932	3.792E-02	hypothetical protein YPO2873
YPO2897	-0.444	7.135E-02	DNA-binding transcriptional regulator IscR
YPO2922	0.672	1.107E-02	transglycosylase
YPO2937	0.839	4.687E-02	hypothetical protein YPO2937
YPO2972	-0.828	9.220E-02	lipoprotein
YPO3010	0.665	2.838E-02	hypothetical protein YPO3010
YPO3048	1.226	4.067E-11	ABC transporter ATP-binding protein
YPO3170	1.286	7.196E-08	nucleotide-binding protein
YPO3207	0.556	3.126E-02	hypothetical protein YPO3207
YPO3208	0.774	6.308E-02	transposase/IS protein
YPO3257	-0.862	9.445E-02	amino acid ABC transporter substrate-binding protein
YPO3387	-1.042	4.758E-07	iron-sulfur cluster insertion protein ErpA
YPO3445	0.470	4.064E-02	hypothetical protein YPO3445
YPO3518	-1.282	3.111E-06	hypothetical protein YPO3518
YPO3556	0.871	3.468E-02	hypothetical protein YPO3556
YPO3617	1.460	9.233E-12	hypothetical protein YPO3617
YPO3618	1.159	1.646E-05	oxidoreductase
YPO3655	1.004	9.815E-08	tRNA-dihydrouridine synthase B
YPO3708	0.617	5.241E-02	hypothetical protein YPO3708
YPO3773	0.702	7.082E-02	transposase/IS protein
YPO3838	-0.577	5.648E-02	hypothetical protein YPO3838
YPO3839	-0.933	2.347E-05	hypothetical protein YPO3839
YPO3957	-1.099	2.424E-02	hypothetical protein YPO3957
YPO3967	1.042	1.991E-05	phosphate transport protein
YPO4005	-0.540	7.129E-02	hemolysin activator protein
YPPCP1.01	0.965	2.580E-02	putative transposase (plasmid)
YPPCP1.02	1.364	7.389E-03	transposase/IS protein (plasmid)
YPPCP1.06	1.028	4.144E-02	hypothetical protein YPPCP1.06 (plasmid)
YPPCP1.09c	0.627	9.445E-02	hypothetical protein YPPCP1.09c (plasmid)
yscK	-0.617	6.981E-03	type III secretion apparatus component (plasmid)
zntA	-1.087	2.797E-05	zinc/cadmium/mercury/lead-transporting ATPase
zwf	-0.484	4.299E-02	glucose-6-phosphate 1-dehydrogenase

"-" indicates a down regulation at the indicated log fold change
