# Supplementary material for: New Insights into Autoinducer-2 Signaling as a Virulence Regulator in a Mouse Model of Pneumonic Plague
Source: mSphere. 2016 Dec 14;1(6):e00342-16. doi: 10.1128/mSphere.00342-16 (PMC5156673; doi:10.1128/mSphere.00342-16)
Supplement: Table S4 [file sph006162209st8.pdf]

Gene Symbol	log fold change	p_{adj}	Genome Annotation
aceB	0.800	5.524E-05	malate synthase
aceE	0.521	3.881E-03	pyruvate dehydrogenase subunit E1
acrA	0.666	1.733E-03	multidrug efflux protein
acrB	0.403	4.907E-02	multidrug efflux protein
astA	0.845	1.588E-02	arginine succinyltransferase
atpI	-1.177	1.491E-06	F0F1 ATP synthase subunit I
bfr	1.458	2.754E-12	bacterioferritin
bioB	-1.023	2.409E-02	biotin synthase
ccmB	-1.061	3.185E-03	heme exporter protein B
ccrB	1.210	1.176E-03	camphor resistance protein CrcB
cheB	-1.447	1.493E-03	chemotaxis-specific methylesterase
clpB3	-1.335	3.572E-03	Clp ATPase
copB	-0.573	1.057E-02	replication protein (plasmid)
cysZ	-0.517	2.265E-02	sulfate transport protein CysZ
ddhC	1.268	2.823E-09	CDP-4-keto-6-deoxy-D-glucose-3-dehydratase
edd	-1.026	1.323E-02	phosphogluconate dehydratase
endA	-0.716	4.795E-02	endonuclease I
fadA	1.289	2.351E-15	3-ketoacyl-CoA thiolase
fadB	1.227	2.486E-16	multifunctional fatty acid oxidation complex subunit alpha
fadE	0.947	1.426E-05	acyl-CoA dehydrogenase
fhuB	-1.008	8.051E-04	iron-hydroxamate transporter permease
fhuC	-1.009	2.086E-02	iron-hydroxamate transporter ATP-binding protein
fldA	-0.772	2.039E-04	flavodoxin FldA
fliG	-1.106	5.370E-03	flagellar motor switch protein G
fliI	-0.954	1.525E-02	flagellum-specific ATP synthase
fliP	-1.016	1.269E-02	flagellar biosynthesis protein FliP
frsA	1.065	2.762E-05	fermentation/respiration switch protein
ftsZ	0.751	1.267E-04	cell division protein FtsZ
fyuA	-1.090	1.381E-04	pesticin/yersiniabactin receptor protein
galF	1.034	6.503E-07	UTP-glucose-1-phosphate uridylyltransferase
glnA	0.420	2.137E-02	glutamine synthetase
gltC	-0.744	8.544E-04	sodium/glutamate symport carrier protein
gptB	1.898	4.143E-07	PTS system mannose-specific transporter subunit IIAB
grxC	1.307	9.375E-04	glutaredoxin 3
hemY	-0.864	5.809E-04	protoheme IX biogenesis protein
hns	-0.435	8.228E-02	global DNA-binding transcriptional dual regulator H-NS
infA	-0.970	3.867E-08	translation initiation factor IF-1
irp4	-0.901	8.090E-02	yersiniabactin biosynthetic protein YbtT

"-" indicates a down regulation at the indicated log fold change

irp5	-1.006	3.447E-02	yersiniabactin siderophore biosynthetic protein
irp8	-1.564	2.299E-04	signal transducer
kdpB	-1.040	2.724E-04	potassium-transporting ATPase subunit B
kdsA	0.856	4.395E-03	2-dehydro-3-deoxyphosphooctonate aldolase
lcrV	0.859	4.507E-09	secreted effector protein (plasmid)
lpxK	-1.005	3.391E-04	tetraacyldisaccharide 4\'-kinase
luxS	-8.909	2.075E-242	S-ribosylhomocysteinase
malK	-1.271	4.482E-03	maltose ABC transporter ATP-binding protein
manY	1.035	8.685E-06	PTS system mannose-specific transporter subunit C
manZ	0.766	7.408E-05	PTS system mannose-specific transporter subunit IID
mda66	-0.944	6.764E-06	modulator of drug activity
mltB	-0.497	6.562E-02	murein hydrolase B
modC	-1.309	3.266E-04	molybdate transporter ATP-binding protein
nagB	1.490	7.038E-09	glucosamine-6-phosphate deaminase
nuoE	0.755	1.893E-03	NADH dehydrogenase subunit E
obgE	0.522	1.007E-03	GTPase ObgE
ompF	-0.750	7.310E-05	porin
oxyR	-0.623	1.357E-02	DNA-binding transcriptional regulator OxyR
pdxA	-1.029	4.099E-06	4-hydroxythreonine-4-phosphate dehydrogenase
phoH	1.311	4.351E-13	hypothetical protein YPO1957
pldA	-0.474	2.152E-02	phospholipase A
proB	0.574	4.344E-02	gamma-glutamyl kinase
psaA	1.940	5.220E-17	pH 6 antigen (antigen 4) (adhesin)
psaE	1.169	1.451E-08	regulatory protein
psaF	1.510	7.281E-10	hypothetical protein YPO1302
psiF	1.127	1.027E-04	starvation-inducible protein
purK	-1.019	1.366E-02	phosphoribosylaminoimidazole carboxylase ATPase subunit
rffG	-1.002	1.277E-05	dTDP-D-glucose-4,6-dehydratase
rhaB	-1.145	4.504E-03	rhamnulokinase
rimI	-0.604	8.919E-02	ribosomal-protein-alanine N-acetyltransferase
rnpB	-1.102	4.993E-03	#N/A
rplA	-0.470	2.260E-02	50S ribosomal protein L1
rpsH	1.148	6.963E-03	30S ribosomal protein S8
rpsO	1.100	1.936E-05	30S ribosomal protein S15
rpsU	-0.369	6.339E-02	30S ribosomal protein S21
sopB	1.164	7.753E-11	plasmid-partitioning protein (plasmid)

"-" indicates a down regulation at the indicated log fold change

ssuC	-1.108	9.657E-03	aliphatic sulfonates transporter permease
sucC	0.686	1.606E-03	succinyl-CoA synthetase subunit beta
surE	-1.088	1.454E-04	stationary phase survival protein SurE
thiD	-1.170	3.881E-03	phosphomethylpyrimidine kinase
tktA	0.715	8.165E-04	transketolase
treC	1.066	5.074E-04	trehalose-6-phosphate hydrolase
trpD	-1.272	3.242E-03	anthranilate phosphoribosyltransferase
trpH	-1.283	3.653E-06	hypothetical protein YPO2211
uppP	-1.028	7.234E-03	undecaprenyl pyrophosphate phosphatase
wrbA	0.624	1.180E-03	TrpR binding protein WrbA
yfcA	-1.301	7.558E-07	hypothetical protein YPO2753
yfeN	-0.386	4.114E-02	hypothetical protein YPO3163
yfiA	1.505	2.852E-15	sigma 54 modulation protein
yggE	1.140	8.361E-07	hypothetical protein YPO0917
yicN	1.057	3.965E-03	hypothetical protein YPO2654
ylpB	0.782	2.233E-06	needle complex inner membrane lipoprotein (plasmid)
ymoA	1.183	5.036E-07	hemolysin expression-modulating protein
ynbB	-1.107	6.578E-03	phosphatidate cytidylyltransferase
yopB	0.540	1.116E-02	secreted effector protein (plasmid)
yopD	0.838	1.233E-06	secreted effector protein (plasmid)
yopH	1.041	5.446E-10	putative secreted protein-tyrosine phosphatase (plasmid)
yopJ	1.135	2.084E-12	targeted effector protein (plasmid)
yopM	1.130	1.306E-09	secreted effector protein (plasmid)
yopO	1.130	1.306E-09	#N/A
yopR	1.566	6.587E-14	secreted protein (plasmid)
yopT	0.759	2.043E-05	Yop targeted effector (plasmid)
YPCD1.07	1.273	6.864E-13	hypothetical protein YPCD1.07 (plasmid)
YPCD1.91n	1.139	4.694E-06	hypothetical protein YPCD1.91n (plasmid)
YPCD1.94	0.675	1.512E-04	putative transposase (plasmid)
YPMT1.07c	-1.157	2.030E-02	putative phage tail protein (plasmid)
YPMT1.13c	-1.027	4.343E-02	hypothetical protein YPMT1.13c (plasmid)
YPMT1.18c	-1.057	9.937E-03	hypothetical protein YPMT1.18c (plasmid)
YPMT1.24c	-1.162	1.473E-02	hypothetical protein YPMT1.24c (plasmid)
YPMT1.26c	-1.379	7.604E-04	hypothetical protein YPMT1.26c (plasmid)
YPMT1.28c	-1.170	1.932E-02	hypothetical protein YPMT1.28c (plasmid)

"-" indicates a down regulation at the indicated log fold change

YPMT1.34A	1.819	3.788E-07	hypothetical protein YPMT1.34A (plasmid)
YPMT1.43c	1.158	6.348E-04	hypothetical protein YPMT1.43c (plasmid)
YPMT1.60c	-1.417	3.166E-03	hypothetical protein YPMT1.60c (plasmid)
YPMT1.61c	-1.600	4.911E-04	antirestriction protein (plasmid)
YPMT1.63c	-1.067	2.817E-02	hypothetical protein YPMT1.63c (plasmid)
YPMT1.76A	-1.165	1.301E-02	hypothetical protein YPMT1.76A (plasmid)
YPO0001	-0.550	4.584E-03	flavodoxin
YPO0013a	0.745	2.724E-04	hypothetical protein YPO0013a
YPO0102	-1.061	6.111E-03	hypothetical protein YPO0102
YPO0237	1.054	2.625E-03	hypothetical protein YPO0237
YPO0267	-1.125	1.955E-02	type III secretion system ATPase
YPO0352	-1.328	1.384E-10	lipoprotein
YPO0368	-0.961	1.634E-04	hypothetical protein YPO0368
YPO0403	-1.194	1.080E-02	PTS system fructose family transporter subunit IIB
YPO0435	-1.488	4.756E-07	Na+ dependent nucleoside transporter family protein
YPO0516	1.003	7.148E-07	hypothetical protein YPO0516
YPO0536	-1.046	7.823E-03	hypothetical protein YPO0536
YPO0622	1.056	2.414E-02	hypothetical protein YPO0622
YPO0647	-0.691	5.458E-03	glycerol-3-phosphate acyltransferase PlsY
YPO0819	1.135	1.239E-07	carbonic anhydrase
YPO0840	-1.108	1.215E-02	hypothetical protein YPO0840
YPO0862	1.333	1.498E-06	hypothetical protein YPO0862
YPO0899	-0.404	6.623E-02	hypothetical protein YPO0899
YPO0904	1.673	1.265E-14	hypothetical protein YPO0904
YPO0936	-1.281	1.080E-06	hypothetical protein YPO0936
YPO0970	-1.025	2.319E-02	hypothetical protein YPO0970
YPO0973	-1.216	9.298E-04	hypothetical protein YPO0973
YPO0976	-1.070	2.948E-02	hypothetical protein YPO0976
YPO0978	-1.544	7.477E-04	hypothetical protein YPO0978
YPO0982	1.198	2.095E-03	lipoprotein
YPO0988	-0.912	3.494E-02	hypothetical protein YPO0988
YPO1011	0.929	2.483E-04	TonB-dependent outer membrane receptor
YPO1033	0.570	5.333E-03	hypothetical protein YPO1033
YPO1061	1.116	2.083E-03	hypothetical protein YPO1061
YPO1064a	-0.961	4.180E-03	hypothetical protein YPO1064a
YPO1090	-1.357	7.464E-04	prophage DNA primase
YPO1097	1.064	3.381E-06	hypothetical protein YPO1097
YPO1158	-1.112	9.422E-05	hypothetical protein YPO1158

"-" indicates a down regulation at the indicated log fold change

YPO1244	-1.212	1.301E-02	hypothetical protein YPO1244
YPO1277	0.620	5.264E-03	cobalamin synthesis protein
YPO1288	-1.151	1.007E-02	D-isomer specific 2-hydroxyacid dehydrogenase family protein
YPO1315	0.876	3.682E-06	hydrolase
YPO1348	-1.062	1.015E-02	hypothetical protein YPO1348
YPO1423	-1.048	1.768E-02	hypothetical protein YPO1423
YPO1446	1.390	3.883E-05	acylphosphatase
YPO1453	1.750	1.560E-07	hypothetical protein YPO1453
YPO1465	-1.023	3.849E-02	hypothetical protein YPO1465
YPO1469	-1.209	1.468E-02	hypothetical protein YPO1469
YPO1470	-1.010	6.350E-03	hypothetical protein YPO1470
YPO1471	-1.386	4.400E-03	ATPase subunit of ATP-dependent protease
YPO1474	1.041	2.192E-03	hypothetical protein YPO1474
YPO1483	-1.067	3.056E-02	hypothetical protein YPO1483
YPO1496	0.933	5.728E-05	heme-binding protein
YPO1534	-1.070	1.892E-02	iron-siderophore transporter membrane permease
YPO1568	0.936	2.801E-05	hypothetical protein YPO1568
YPO1649	0.461	2.368E-02	hypothetical protein YPO1649
YPO1669	1.284	1.723E-03	hypothetical protein YPO1669
YPO1694	1.235	2.165E-07	hypothetical protein YPO1694
YPO1707	1.279	2.921E-04	fimbrial protein
YPO1747	-1.022	6.398E-06	hypothetical protein YPO1747
YPO1788	1.529	2.813E-07	acyl carrier protein
YPO1818	-1.012	2.086E-02	hypothetical protein YPO1818
YPO1975	1.030	3.036E-03	hypothetical protein YPO1975
YPO2031	-1.083	2.365E-02	binding-protein-dependent transporter membrane protein
YPO2051	0.921	2.724E-04	hypothetical protein YPO2051
YPO2187	-0.666	6.059E-03	dsDNA-mimic protein
YPO2277	0.602	8.760E-02	hypothetical protein YPO2277
YPO2331	0.957	1.735E-03	lipoprotein
YPO2379	0.610	6.564E-03	N-ethylmaleimide reductase
YPO2385	0.827	1.477E-04	hypothetical protein YPO2385
YPO2653	1.045	3.428E-04	hypothetical protein YPO2653
YPO2683	1.061	1.530E-02	hypothetical protein YPO2683
YPO2792	1.479	6.089E-04	hypothetical protein YPO2792
YPO2806	1.367	1.918E-08	aldo/keto reductase
YPO2822	1.165	1.557E-03	hypothetical protein YPO2822
YPO2842	-1.001	3.388E-02	ABC transporter permease
YPO2864	1.153	2.158E-02	hypothetical protein YPO2864
YPO2923	1.283	2.408E-04	tRNA-specific adenosine deaminase
YPO3137	1.401	1.941E-09	hypothetical protein YPO3137
YPO3150	0.668	1.421E-03	queuosine biosynthesis protein QueC
YPO3348	1.575	5.950E-11	transcriptional regulator

"-" indicates a down regulation at the indicated log fold change

YPO3476	-1.206	5.470E-07	acetyltransferase
YPO3498	-0.858	5.916E-05	hypothetical protein YPO3498
YPO3549	-1.327	2.832E-07	hypothetical protein YPO3549
YPO3613	-1.199	1.079E-02	Rhs accessory genetic element
YPO3699	-0.370	2.781E-02	hypothetical protein YPO3699
YPO3799	-1.055	2.157E-02	hypothetical protein YPO3799
YPO3801	-1.013	4.571E-02	hypothetical protein YPO3801
YPO3828	0.605	1.651E-02	hypothetical protein YPO3828
YPO3885	1.114	1.435E-03	hypothetical protein YPO3885
YPO3902	-1.108	5.710E-03	magnesium chelatase family protein
YPO3904	1.243	1.306E-05	transcriptional regulator HdfR
YPO3956	0.723	2.100E-02	hypothetical protein YPO3956
YPO3963	-1.316	4.467E-03	sugar transport system permease
YPO3965	-0.549	4.701E-02	hybrid two-component system regulatory protein
yscA	1.367	4.912E-12	hypothetical protein YPCD1.50 (plasmid)
yscB	0.525	2.281E-02	type III secretion apparatus component (plasmid)
yscC	0.440	1.128E-02	outer membrane secretin precursor (plasmid)
yscD	0.836	5.383E-07	virulence protein (plasmid)
yscG	1.139	1.201E-09	type III secretion apparatus component (plasmid)
yscL	1.139	1.201E-09	type III secretion system protein (plasmid)
yscO	0.450	9.335E-03	type III secretion apparatus component (plasmid)
yscP	0.484	3.715E-03	type III secretion apparatus component (plasmid)
yscR	0.593	6.103E-05	type III secretion system protein (plasmid)
yscS	0.521	4.370E-03	needle complex export protein (plasmid)
yscT	0.508	1.843E-02	needle complex export protein (plasmid)
yscU	0.653	9.754E-05	needle complex export protein (plasmid)
yscV	0.506	9.350E-04	low calcium response protein D (plasmid)
yscX	0.434	1.137E-01	hypothetical protein YPCD1.36c (plasmid)
yscY	-0.687	1.140E-01	hypothetical protein YPCD1.35c (plasmid)
zipA	0.682	1.141E-01	cell division protein ZipA

"-" indicates a down regulation at the indicated log fold change
