# Supplementary material for: New Insights into Autoinducer-2 Signaling as a Virulence Regulator in a Mouse Model of Pneumonic Plague
Source: mSphere. 2016 Dec 14;1(6):e00342-16. doi: 10.1128/mSphere.00342-16 (PMC5156673; doi:10.1128/mSphere.00342-16)
Supplement: Table S5 [file sph006162209st9.pdf]

Gene Symbol	log fold change	p_{adj}	Genome Annotation
aceA	1.039	1.698E-07	isocitrate lyase
aceK	0.527	4.180E-03	bifunctional isocitrate dehydrogenase kinase/phosphatase protein
acs	0.505	3.935E-03	acetyl-CoA synthetase
agaZ	-0.786	9.286E-03	tagatose 6-phosphate kinase
ahpC	0.604	1.439E-03	alkyl hydroperoxide reductase
apt	-1.163	2.762E-05	adenine phosphoribosyltransferase
aroL	-0.703	1.390E-03	shikimate kinase II
atpD	0.562	2.084E-03	ATP synthase F0F1 subunit beta
bioF	-0.962	2.930E-02	8-amino-7-oxononanoate synthase
btuC	0.940	9.710E-05	vtamin B12-transporter permease
btuD	1.491	1.040E-08	vitamin B12-transporter ATPase
cafA	0.504	1.861E-02	ribonuclease G
clpP	0.941	6.145E-05	ATP-dependent Clp protease proteolytic subunit
cls	-1.077	1.771E-08	cardiolipin synthetase
creA	0.584	2.217E-03	hypothetical protein YPO0457
cyaB	0.762	8.966E-05	adenylate cyclase
cydA	0.666	3.753E-04	cytochrome D ubiquinol oxidase subunit I
cydB	0.438	4.782E-02	cytochrome D ubiquinol oxidase subunit II
cyoC	0.496	4.250E-03	cytochrome o ubiquinol oxidase subunit III
cysK	-0.502	5.519E-03	cysteine synthase A
cysM	-0.649	3.036E-03	cysteine synthase B
cysN	-0.585	9.623E-02	sulfate adenylyltransferase subunit 1
cysT	-0.841	6.566E-04	sulfate/thiosulfate transporter subunit
cysW	-0.994	1.811E-04	sulfate/thiosulfate transporter permease
dadA	-1.291	8.502E-15	D-amino acid dehydrogenase small subunit
dapF	-1.145	1.024E-05	diaminopimelate epimerase
dcrB	0.820	1.027E-04	hypothetical protein YPO3823
ddhD	0.905	1.035E-02	CDP-6-deoxy-delta-3,4-glucoseen reductase
deoC	-0.574	1.704E-02	deoxyribose-phosphate aldolase
dsbB	-1.182	2.790E-09	disulfide bond formation protein B
elaB	1.243	7.757E-05	hypothetical protein YPO2531
engA	0.376	4.836E-02	GTP-binding protein EngA
fadJ	0.538	1.017E-03	multifunctional fatty acid oxidation complex subunit alpha
flgJ	-0.814	4.296E-02	peptidoglycan hydrolase
fliH	-0.767	5.874E-02	flagellar assembly protein H
fliN	-0.829	9.382E-02	flagellar switch protein

"-" indicates a down regulation at the indicated log fold change

frdB	0.721	2.489E-03	fumarate reductase iron-sulfur subunit
ftsW	0.557	1.366E-02	cell division protein FtsW
gcsH	0.540	4.493E-02	glycine cleavage system protein H
gdhA	-0.441	3.749E-02	glutamate dehydrogenase
glnB	0.404	5.938E-02	nitrogen regulatory protein P-II 1
glnH	0.295	9.752E-02	glutamine ABC transporter substrate-binding protein
glnP	1.683	2.126E-13	glutamine ABC transporter permease
glnQ	1.449	1.671E-12	glutamine ABC transporter ATP-binding protein
glyA	0.490	3.028E-02	serine hydroxymethyltransferase
gnd	0.418	1.219E-02	6-phosphogluconate dehydrogenase
gpmA	0.712	2.995E-05	phosphoglyceromutase
grxA	0.798	2.991E-06	glutaredoxin
gsrA	0.722	2.534E-05	serine endoprotease
guaA	0.681	1.353E-02	GMP synthase
hcaT	-0.973	1.249E-03	3-phenylpropionic acid transporter
hflB	0.622	1.103E-04	ATP-dependent metalloprotease
hflC	0.414	4.702E-02	FtsH protease regulator HflC
hisJ	0.416	1.598E-02	histidine-binding periplasmic protein
hmwA	-0.396	9.433E-02	adhesin
hofQ	-0.307	9.582E-02	porin
hpaX	-1.041	2.193E-04	4-hydroxyphenylacetate permease
hslR	-0.674	1.098E-02	heat shock protein 15
hupA	0.481	6.079E-02	transcriptional regulator HU subunit alpha
icdA	0.759	1.811E-04	isocitrate dehydrogenase
ihfB	1.114	2.313E-08	integration host factor subunit beta
infB	0.418	1.804E-02	translation initiation factor IF-2
infC	1.767	2.558E-12	translation initiation factor IF-3
irp1	-1.719	9.119E-07	yersiniabactin biosynthetic protein
irp2	-1.081	7.403E-04	yersiniabactin biosynthetic protein
irp3	-1.375	4.403E-03	yersiniabactin biosynthetic protein YbtU
irp4	-0.901	8.090E-02	yersiniabactin biosynthetic protein YbtT
irp5	-1.006	3.447E-02	yersiniabactin siderophore biosynthetic protein
irp6	-0.873	4.437E-02	lipoprotein inner membrane ABC transporter
irp7	-0.961	2.817E-02	ABC transporter permease
irp8	-1.564	2.299E-04	signal transducer
ispG	0.534	1.817E-02	4-hydroxy-3-methylbut-2-en-1-yl diphosphate synthase
katA	0.415	3.099E-02	catalase
ksgA	1.241	1.903E-05	dimethyladenosine transferase

"-" indicates a down regulation at the indicated log fold change

lcrG	0.835	6.349E-09	low calcium response protein G (plasmid)
livG	-0.723	5.525E-02	leucine/isoleucine/valine transporter ATP-binding protein
livM	-0.630	7.328E-03	leucine/isoleucine/valine transporter permease
lplA	1.579	4.750E-24	lipoate-protein ligase A
lpxC	0.521	1.740E-03	UDP-3-O
mipB	-1.088	4.479E-04	fructose-6-phosphate aldolase
mobA	0.976	8.764E-07	molybdopterin-guanine dinucleotide biosynthesis protein MobA
mobB	0.773	9.873E-04	molybdopterin-guanine dinucleotide biosynthesis protein B
mscL	0.612	8.228E-04	large-conductance mechanosensitive channel
mtta1	0.641	6.114E-04	#N/A
murB	0.449	1.560E-02	UDP-N-acetylenolpyruvoylglucosamine reductase
nagE	1.109	4.581E-08	PTS system N-acetylglucosamine-specific transporter subunit IIABC
nhaB	-1.284	1.417E-11	sodium/proton antiporter
nlpC	0.718	5.298E-03	lipoprotein
nqrF	0.540	2.765E-02	Na(+)-translocating NADH-quinone reductase subunit F
nudG	-1.433	5.901E-05	pyrimidine (deoxy)nucleoside triphosphate pyrophosphohydrolase
ompR	0.409	2.888E-02	osmolarity response regulator
ompW	0.433	7.787E-02	outer membrane protein W
ompX	1.014	6.365E-09	outer membrane protein X
oppD	-0.802	5.012E-05	oligopeptide transporter ATP-binding component
oppF	-1.172	1.010E-06	oligopeptide transport ATP-binding protein
pabB	-0.624	1.598E-02	para-aminobenzoate synthase component I
pal	0.744	3.316E-04	peptidoglycan-associated outer membrane lipoprotein
pbpG	1.073	1.060E-04	D-alanyl-D-alanine endopeptidase
pepD	0.368	3.133E-02	aminoacyl-histidine dipeptidase
pfkA	1.046	3.269E-05	6-phosphofructokinase
pheS	0.805	3.318E-06	phenylalanyl-tRNA synthetase subunit alpha
phrB	-0.918	8.188E-04	deoxyribodipyrimidine photolyase
pla	0.579	5.297E-02	outer membrane protease (plasmid)
pmrF	1.517	8.834E-21	undecaprenyl phosphate 4-deoxy-4-formamido-L-arabinose transferase
pncA	-1.043	1.677E-05	nicotinamidase/pyrazinamidase

"-" indicates a down regulation at the indicated log fold change

prc	0.550	6.102E-04	carboxy-terminal protease
psaC	-0.724	2.875E-03	outer membrane usher protein PsaC
pspF	-0.731	7.076E-04	phage shock protein operon transcriptional activator
pspG	-1.692	1.630E-05	phage shock protein G
pst	1.093	7.453E-03	pesticin (plasmid)
ptsI	0.952	2.219E-05	phosphoenolpyruvate-protein phosphotransferase
rhaA	-1.239	4.257E-03	L-rhamnose isomerase
ribE	0.600	8.188E-04	riboflavin synthase subunit alpha
ribH	0.429	1.892E-02	6,7-dimethyl-8-ribityllumazine synthase
rlpA	0.403	8.737E-02	rare lipoprotein A
rnk	0.783	5.062E-04	nucleoside diphosphate kinase regulator
rplS	1.211	5.847E-05	50S ribosomal protein L19
rplT	1.757	5.262E-05	50S ribosomal protein L20
rpoN	0.776	2.658E-05	RNA polymerase factor sigma-54
rpoZ	1.111	2.488E-04	DNA-directed RNA polymerase subunit omega
rpsD	0.856	2.154E-03	30S ribosomal protein S4
rth	-0.491	2.227E-02	undecaprenyl pyrophosphate synthase
rumB	-0.905	9.024E-03	23S rRNA methyluridine methyltransferase
selD	-1.626	1.057E-18	selenophosphate synthetase
sodB	0.914	7.837E-07	superoxide dismutase
spf	1.185	8.773E-03	#N/A
sppA	-0.571	1.981E-03	protease 4
ssuB	-0.733	7.285E-02	aliphatic sulfonates transporter ATP-binding protein
surA	0.427	3.363E-02	peptidyl-prolyl cis-trans isomerase SurA
tam	1.299	6.118E-08	trans-aconitate 2-methyltransferase
tatE	-0.487	7.032E-02	twin-arginine translocation protein TatA
tauC	-0.945	5.332E-02	taurine transporter subunit
tauD	-0.644	4.019E-02	taurine dioxygenase
thrS	1.075	1.749E-05	threonyl-tRNA synthetase
tonB	-1.690	9.903E-18	transport protein TonB
topB	-1.048	3.311E-11	DNA topoisomerase III
tpiA	1.390	4.471E-08	triosephosphate isomerase
tppB	-0.544	4.592E-03	tripeptide transporter permease
trkH	-0.523	7.234E-03	potassium transporter
trmB	0.487	6.682E-02	tRNA (guanine-N(7)-)-methyltransferase
ubiH	-0.656	5.077E-03	2-octaprenyl-6-methoxyphenyl hydroxylase

"-" indicates a down regulation at the indicated log fold change

ubiX	-0.368	8.126E-02	3-octaprenyl-4-hydroxybenzoate carboxy-lyase
ugpC	-0.795	4.740E-03	glycerol-3-phosphate transporter ATP-binding protein
uvrB	-0.309	9.692E-02	excinuclease ABC subunit B
wbyH	0.943	5.736E-06	hypothetical protein YPO3111
wbyK	0.609	7.387E-02	mannosyltransferase
xthA	-1.281	1.490E-09	exonuclease III
yapC	-0.928	1.727E-04	autotransporter protein
ybeX	0.385	4.944E-02	hypothetical protein YPO2617
ybjR	0.441	4.360E-02	#N/A
ydeN	1.081	2.393E-11	sulfatase
yebY	0.576	1.313E-02	hypothetical protein YPO1786
yecS	-0.780	2.231E-03	amino-acid ABC transporter permease
yeiB	-0.568	2.220E-02	hypothetical protein YPO1506
yfeA	1.965	3.266E-40	substrate-binding protein
yfeB	1.915	8.948E-35	ATP-binding transport protein
yfeC	1.357	7.281E-10	chelated iron transport system membrane protein
yfeD	1.188	4.872E-10	chelated iron transport system membrane protein
yfeE	2.343	2.143E-42	yfeABCD locus regulator
ygeD	-0.995	1.944E-03	lysophospholipid transporter LplT
yhbG	0.597	1.731E-03	ABC transporter ATP-binding protein YhbG
yidE	-0.648	1.498E-02	hypothetical protein YPO4083
ylaC	0.819	9.805E-07	hypothetical protein YPO1652
ypeI	2.192	5.649E-23	N-acylhomoserine lactone synthase
ypeR	2.140	1.284E-24	quorum-sensing transcriptional activator YpeR
YPMT1.03c	-0.701	2.408E-02	tail fiber assembly protein G (plasmid)
YPMT1.11c	-1.623	3.547E-05	hypothetical protein YPMT1.11c (plasmid)
YPMT1.22c	-1.388	2.450E-03	hypothetical protein YPMT1.22c (plasmid)
YPMT1.32	-1.183	5.539E-04	putative lipoprotein (plasmid)
YPMT1.33	-1.170	1.490E-03	putative transcriptional regulator (plasmid)
YPMT1.35c	-1.517	3.050E-05	hypothetical protein YPMT1.35c (plasmid)
YPMT1.45c	1.201	1.329E-02	hypothetical protein YPMT1.45c (plasmid)
YPMT1.46c	0.874	7.318E-02	hypothetical protein YPMT1.46c (plasmid)
YPMT1.59c	-1.801	5.220E-05	putative DNA-binding protein (plasmid)

"-" indicates a down regulation at the indicated log fold change

YPMT1.71	-1.005	1.118E-06	hypothetical protein YPMT1.71 (plasmid)
YPMT1.72c	-0.812	1.722E-04	hypothetical protein YPMT1.72c (plasmid)
YPMT1.75c	-1.164	1.168E-02	reverse transcriptase (plasmid)
YPO0007	0.619	8.306E-04	D-ribose pyranase
YPO0014	0.635	1.282E-03	serine/threonine protein kinase
YPO0027	0.527	1.181E-02	phosphatase
YPO0032	-0.837	1.710E-06	hypothetical protein YPO0032
YPO0034	-0.900	1.234E-02	membrane permease
YPO0128	-1.050	2.485E-03	gluconate periplasmic binding protein
YPO0147	-1.185	4.057E-04	hypothetical protein YPO0147
YPO0148	-1.459	5.842E-04	hypothetical protein YPO0148
YPO0196	0.757	3.686E-04	DNA-binding protein
YPO0199	0.890	1.630E-08	sulfur transfer complex subunit TusB
YPO0391	0.700	1.371E-03	modification methylase
YPO0392	1.050	3.453E-04	hypothetical protein YPO0392
YPO0397	-1.125	8.544E-04	hypothetical protein YPO0397
YPO0405	-0.950	1.296E-03	phosphoenolpyruvate-protein phosphotransferase
YPO0498	0.957	7.921E-07	hypothetical protein YPO0498
YPO0502	0.786	1.378E-02	hypothetical protein YPO0502
YPO0599	-0.565	3.669E-03	adhesin
YPO0651	0.673	1.539E-04	signal transduction protein
YPO0659	-0.677	7.753E-02	hypothetical protein YPO0659
YPO0806	1.044	9.481E-03	prepilin peptidase
YPO0820	-0.887	7.479E-02	hypothetical protein YPO0820
YPO0912	0.824	1.862E-05	Z-ring-associated protein
YPO0919	0.635	3.617E-03	hypothetical protein YPO0919
YPO1001	-0.813	3.373E-03	integral membrane efflux protein
YPO1052	0.468	1.981E-03	outer membrane protein assembly factor YaeT
YPO1064	0.881	2.246E-04	Rho-binding antiterminator
YPO1072	1.003	1.913E-04	DL-methionine transporter permease
YPO1091	-0.689	1.366E-02	prophage protein
YPO1179	0.767	1.488E-03	hypothetical protein YPO1179
YPO1201	-0.359	9.032E-02	amino acid decarboxylase
YPO1255	0.797	9.842E-05	hypothetical protein YPO1255
YPO1257	0.747	6.594E-03	hypothetical protein YPO1257
YPO1316	-0.628	3.200E-02	iron/ascorbate oxidoreductase family protein
YPO1318	-1.197	5.867E-04	ABC transporter ATP-binding protein
YPO1364	0.486	3.049E-02	macrolide transporter subunit MacA
YPO1401	0.701	3.186E-03	hypothetical protein YPO1401
YPO1450d	-0.522	6.181E-02	hypothetical protein YPO1450d
YPO1490	0.751	3.592E-03	hypothetical protein YPO1490
YPO1492	0.759	2.158E-02	hypothetical protein YPO1492

"-" indicates a down regulation at the indicated log fold change

YPO1500	1.277	1.320E-04	hypothetical protein YPO1500
YPO1575	0.905	1.213E-05	hypothetical protein YPO1575
YPO1614	-0.779	7.901E-02	hypothetical protein YPO1614
YPO1643	0.731	8.014E-04	hypothetical protein YPO1643
YPO1693	0.414	5.957E-02	hypothetical protein YPO1693
YPO1718	1.039	8.337E-09	hypothetical protein YPO1718
YPO1736	1.603	2.242E-14	hypothetical protein YPO1736
YPO1887	1.124	2.068E-03	hypothetical protein YPO1887
YPO1918	-0.611	3.565E-02	pili assembly chaperone
YPO1925	0.761	8.775E-06	two-component response regulator
YPO1931	0.698	1.312E-02	hypothetical protein YPO1931
YPO1933	-0.606	4.856E-02	dicarboxylic acid hydrolase
YPO1989	0.527	2.042E-02	hypothetical protein YPO1989
YPO2039	0.942	1.775E-04	hypothetical protein YPO2039
YPO2055	0.695	4.619E-04	hypothetical protein YPO2055
YPO2068	-0.939	1.886E-03	hypothetical protein YPO2068
YPO2082	0.993	2.731E-04	hypothetical protein YPO2082
YPO2095	1.793	7.315E-07	hypothetical protein YPO2095
YPO2123	-0.884	5.715E-02	phage minor tail protein
YPO2126	0.352	8.025E-02	hypothetical protein YPO2126
YPO2127	0.412	9.893E-02	phage-like membrane protein
YPO2128	0.994	7.858E-04	phage-like lipoprotein
YPO2133	-0.639	2.959E-02	hypothetical protein YPO2133
YPO2138	-1.656	3.282E-05	aminotransferase
YPO2139	-1.793	1.201E-04	hypothetical protein YPO2139
YPO2140	-1.338	3.514E-12	hypothetical protein YPO2140
YPO2145	-0.968	2.787E-08	SpoVR family protein
YPO2149	-0.985	5.412E-09	hypothetical protein YPO2149
YPO2150	-0.546	9.405E-02	LysR family transcriptional regulator
YPO2151	-0.941	8.150E-05	hypothetical protein YPO2151
YPO2152	-1.018	1.354E-07	hypothetical protein YPO2152
YPO2155	-0.626	3.444E-04	hypothetical protein YPO2155
YPO2156	-0.871	6.446E-07	hypothetical protein YPO2156
YPO2163	-1.906	8.821E-15	hypothetical protein YPO2163
YPO2169	-2.744	3.420E-16	LysR family transcriptional regulator
YPO2171	-0.877	3.413E-07	formyltetrahydrofolate deformylase
YPO2172	-1.767	9.037E-17	hypothetical protein YPO2172
YPO2189	-0.700	3.047E-02	hypothetical protein YPO2189
YPO2192	-1.422	6.844E-08	hypothetical protein YPO2192
YPO2202	0.907	1.163E-05	lipoprotein
YPO2231	1.581	1.022E-04	hypothetical protein YPO2231
YPO2246	-0.863	1.045E-02	Na(+)-translocating NADH-quinone reductase subunit E
YPO2271	0.877	2.944E-04	hypothetical protein YPO2271
YPO2272	0.850	6.710E-04	hypothetical protein YPO2272
YPO2321	-0.538	6.245E-02	hypothetical protein YPO2321
YPO2398	1.406	9.116E-17	murein L,D-transpeptidase

"-" indicates a down regulation at the indicated log fold change

YPO2399	0.816	3.659E-03	cysteine desufuration protein SufE
YPO2406	0.979	7.146E-06	hypothetical protein YPO2406
YPO2407	1.191	1.702E-10	hypothetical protein YPO2407
YPO2408	1.217	1.490E-09	hypothetical protein YPO2408
YPO2410	0.963	1.259E-05	hypothetical protein YPO2410
YPO2419	1.449	3.844E-15	hypothetical protein YPO2419
YPO2420	1.865	8.667E-32	bifunctional UDP-glucuronic acid decarboxylase/UDP-4-amino-4-deoxy-L-arabinose formyltransferase
YPO2422	1.270	4.132E-15	UDP-4-amino-4-deoxy-L-arabinose--oxoglutarate aminotransferase
YPO2426	1.728	4.474E-18	hypothetical protein YPO2426
YPO2444	0.986	2.341E-06	hypothetical protein YPO2444
YPO2446	1.382	3.325E-08	2-deoxyglucose-6-phosphatase
YPO2449	1.640	2.667E-30	LuxR family transcriptional regulator
YPO2451	1.017	2.790E-09	hypothetical protein YPO2451
YPO2452	0.643	1.952E-02	hypothetical protein YPO2452
YPO2455	1.222	6.262E-06	hypothetical protein YPO2455
YPO2458	1.003	3.033E-05	LysR family transcriptional regulator
YPO2459	1.022	3.078E-03	transporter protein
YPO2460	0.959	2.281E-02	hypothetical protein YPO2460
YPO2461	0.823	2.953E-04	oxidoreductase
YPO2462	1.731	2.195E-10	hypothetical protein YPO2462
YPO2463	0.940	3.147E-05	hypothetical protein YPO2463
YPO2464	0.990	1.513E-04	hypothetical protein YPO2464
YPO2467	1.222	3.567E-10	hypothetical protein YPO2467
YPO2468	0.616	1.070E-02	hypothetical protein YPO2468
YPO2470	0.773	4.334E-02	hypothetical protein YPO2470
YPO2471	1.165	8.950E-07	hypothetical protein YPO2471
YPO2473	1.184	8.685E-06	hypothetical protein YPO2473
YPO2476	1.714	1.524E-10	sugar ABC transporter permease
YPO2477	0.799	2.987E-03	solute-binding protein
YPO2482	1.629	3.242E-09	hypothetical protein YPO2482
YPO2484	2.004	7.837E-07	hypothetical protein YPO2484
YPO2485	1.235	6.900E-07	hypothetical protein YPO2485
YPO2494	1.234	1.869E-05	transporter
YPO2495	1.118	2.762E-04	hypothetical protein YPO2495
YPO2496	1.452	6.602E-09	tartrate dehydrogenase
YPO2497	0.686	2.292E-02	LysR family transcriptional regulator
YPO2503	1.041	1.664E-03	hypothetical protein YPO2503
YPO2504	2.001	1.354E-14	hypothetical protein YPO2504
YPO2505	1.135	6.577E-05	hypothetical protein YPO2505
YPO2515	1.269	7.673E-12	chemotactic transducer
YPO2542	1.092	3.696E-06	hypothetical protein YPO2542
YPO2606	0.467	1.804E-02	hypothetical protein YPO2606
YPO2611	0.740	6.221E-05	hypothetical protein YPO2611
YPO2675	1.283	4.061E-10	voltage-gated potassium channel

"-" indicates a down regulation at the indicated log fold change

YPO2701	-1.174	7.586E-04	hypothetical protein YPO2701
YPO2705	0.979	1.349E-06	autonomous glycyl radical cofactor GrcA
YPO2745	1.703	1.996E-05	hypothetical protein YPO2745
YPO2761	-1.020	4.474E-03	hypothetical protein YPO2761
YPO2795	0.790	2.484E-03	hypothetical protein YPO2795
YPO2820	0.979	7.398E-05	hypothetical protein YPO2820
YPO2840	-0.930	1.057E-02	chaperone
YPO2950	-0.695	1.473E-02	fimbrial protein
YPO2954	0.878	9.698E-06	hypothetical protein YPO2954
YPO2963	0.812	8.397E-03	hypothetical protein YPO2963
YPO3121	1.187	1.626E-05	hypothetical protein YPO3121
YPO3136	1.129	4.155E-07	hypothetical protein YPO3136
YPO3149	-0.736	8.544E-04	hypothetical protein YPO3149
YPO3309	-0.524	4.835E-02	hypothetical protein YPO3309
YPO3414	0.364	6.399E-02	hypothetical protein YPO3414
YPO3528	0.733	2.515E-04	hypothetical protein YPO3528
YPO3548	-0.660	1.211E-03	hypothetical protein YPO3548
YPO3564	-0.821	1.053E-04	hypothetical protein YPO3564
YPO3580	0.555	8.188E-04	lipopolysaccharide transport periplasmic protein LptA
YPO3681	-2.505	2.323E-41	insecticial toxin
YPO3682	-2.900	7.471E-31	LysR family transcriptional regulator
YPO3694	0.873	2.354E-05	cytochrome
YPO3744	-1.503	5.886E-04	hypothetical protein YPO3744
YPO3791	-0.851	1.727E-02	hypothetical protein YPO3791
YPO3821	-1.469	1.316E-06	sulfur transfer protein SirA
YPO3874	1.261	1.527E-07	hypothetical protein YPO3874
YPO3880	-0.490	5.623E-02	hypothetical protein YPO3880
YPO3908	0.906	2.758E-05	periplasmic protein
YPO3944	-0.459	4.389E-03	invasin
YPO3948	1.259	8.579E-04	hypothetical protein YPO3948
YPO3991	0.610	1.176E-04	insulinase family protease
YPO4050	1.083	2.024E-04	hypothetical protein YPO4050
YPO4081	1.053	3.849E-02	hypothetical protein YPO4081
YPO4109	-0.726	3.669E-03	amino acid transport system permease
YPO4110	-1.310	3.702E-07	ABC transporter permease
YPPCP1.08c	0.684	5.809E-04	putative transcriptional regulator (plasmid)
YPt_02	-1.132	2.412E-02	#N/A
YPt_03	-0.790	4.205E-02	#N/A
YPt_29	-1.535	3.083E-07	#N/A
YPt_59	-1.535	2.147E-06	#N/A
YPt_63	-1.679	1.791E-06	#N/A
YPt_65	-0.986	9.853E-06	#N/A
YPt_70	-0.850	9.324E-02	#N/A
yspI	-0.769	1.476E-01	N-acylhomoserine lactone synthase

"-" indicates a down regulation at the indicated log fold change
